# Treatment Effects of Ischemic Stroke by Berberine, Baicalin, and Jasminoidin from Huang-Lian-Jie-Du-Decoction (HLJDD) Explored by an Integrated Metabolomics Approach

**DOI:** 10.1155/2017/9848594

**Published:** 2017-08-14

**Authors:** Qian Zhang, Xiaowei Fu, Junsong Wang, Minghua Yang, Lingyi Kong

**Affiliations:** ^1^Jiangsu Key Laboratory of Bioactive Natural Product Research and State Key Laboratory of Natural Medicines, Department of Natural Medicinal Chemistry, China Pharmaceutical University, 24 Tong Jia Xiang, 210009 Nanjing, China; ^2^Center for Molecular Metabolism, Nanjing University of Science and Technology, 200 Xiao Ling Wei Street, 210094 Nanjing, China

## Abstract

Berberine, baicalin, and jasminoidin were major active ingredients of Huang-Lian-Jie-Du-Decoction (HLJDD), a famous prescription of traditional Chinese medicine (TCM), which has been used for the treatment of ischemic stroke. The aim of the present study was to classify their roles in the treatment effects of ischemic stroke. A rat model of middle cerebral artery occlusion (MCAO) was constructed to mimic ischemic stroke and treatment effects of berberine, baicalin, and jasminoidin, and HLJDD was assessed by neurologic deficit scoring, infarct volume, histopathology, immunohistochemistry, biochemistry, quantitative real-time polymerase chain reaction (qRT-PCR), and Western blotting. In addition, the ^1^H NMR metabolomics approach was used to assess the metabolic profiles, which combined with correlation network analysis successfully revealed metabolic disorders in ischemic stroke concerning the treatment of the three principal compounds from HLJDD for the first time. The combined results suggested that berberine, baicalin, and jasminoidin are responsible for the effectiveness of HLJDD on the treatment of ischemic stroke by amelioration of abnormal metabolism and regulation of oxidative stress, neuron autophagy, and inflammatory response. This integrated metabolomics approach showed its potential in understanding the function of complex formulae and clarifying the role of its components in the overall treatment effects.

## 1. Introduction

Stroke is one of the leading causes of death and disability worldwide, of which ischemic stroke accounts for approximately 85% [1, 2]. Over the past few years, the pharmaceutical industry has seen a shift from the search for “magic bullets” that specifically target a single disease-causing molecule to the pursuit of combination therapies that comprise more than one active ingredients to treat complex illnesses such as ischemic stroke. This shift coincides with the tested advocacy of traditional Chinese medicine (TCM) to utilize combinatorial therapeutic strategies [3].

Composed of *Rhizoma coptidis*, *Radix scutellariae*, *Cortex phellodendri*, and *Fructus gardenia*, Huang-Lian-Jie-Du-Decoction (HLJDD) is a famous multiherb TCM formula that has been used for the treatment of cerebrovascular diseases and ischemic stroke in the clinical practice of TCM [4–6]. Currently, considerable studies have been performed aiming to understand the pharmacological effects of HLJDD on ischemia-induced brain damage [7–9]. Our previous study showcased good efficacy of HLJDD in the treatment of ischemic stroke [10, 11]. However, the role of its principals in the treatment of stroke remains largely unknown.

Alkaloids (e.g., berberine, palmatine, and coptisine), flavonoids (e.g., baicalin, baicalein, wogonoside, and wogonin), and iridoids (e.g., geniposide and shanzhiside) are major active components of HLJDD, and among them, berberine (Ber), baicalin (Bai), and jasminoidin (Jas) are the three major ingredients [12, 13]. HLJDD extracts, as well as its components, have protective effects against ischemic stroke [14, 15]. Berberine, the main constituent of *Rhizoma coptidis* and *Cortex phellodendri*, is an isoquinoline alkaloid with a long history of medicinal use in both Ayurvedic and Chinese medicine. Recently, berberine has been shown to possess potent neuroprotective effects against ischemic damage [15, 16]. Baicalin, the main ingredient of *Radix scutellariae* (one of the most common herbs that are used as stroke therapeutic drugs in TCM [17]), directly protects neuronal cells against various neurotoxic stimuli and ischemia-reperfusion injury [18]. Jasminoidin is the effective component of *Fructus gardenia*, which enhances the viability of neurons, prompts neurite growth, and attenuates neuronal death in modelled ischemic environment [19].

In this study, a middle cerebral artery occlusion (MCAO) rat model was established, which could faithfully mimic the pathophysiological changes found in stroke patients [20]. The present study was conducted to explore the role of the three main active components from HLJDD on brain injury after focal cerebral ischemia in a rat model. Furthermore, we investigated the underlying mechanisms of the three active components on ischemic brain injury using metabolomics and molecular biology methods.

## 2. Materials and Methods

### 2.1. Chemicals and Reagents

Details on berberine (Ber), baicalin (Bai), jasminoidin (Jas) and preparation of HLJDD extract and other materials used can be found in Supplementary Information available online at https://doi.org/10.1155/2017/9848594.

### 2.2. Quantitative Analysis by HPLC

For quantitative analysis, a standard solution for berberine, baicalin, and jasminoidin was prepared in methanol. The calibration curves were constructed by plotting the peak area versus the corresponding concentration levels. The amounts of berberine, baicalin, and jasminoidin in HLJDD extract were quantified. The standard and sample solutions were both filtered through a 0.22 *μ*m membrane filter prior to injection to the HPLC system.

The HPLC analyses were performed on an Agilent 1290 HPLC instrument (Agilent Technologies Corporation, Santa Clara, CA, USA). Chromatographic separation was performed on a Shimadzu VP-ODS column (250 × 4.6 mm ID, 5 *μ*m particle size; Shimadzu, Kyoto, Japan) with a solvent flow rate of 1 ml/min at a temperature of 30°C. The mobile phase was composed of 10 mmol/l ammonium acetate titrated with acetic acid to pH 3.0 (A) and acetonitrile (B). The solvent gradient adopted was as follows: 0–4 min, 10% B; 4–15 min, 10–26% B; 15–27 min, 26–28% B; 27–35 min, 28–70% B; 35–55 min, 70–90% B; and 55–60 min, 90% B [21]. An 8 min postrun time back to the initial mobile phase composition was used after each analysis. The injection volume was 5 *μ*l and was detected at 238 nm, 254 nm, and 280 nm. See Supplementary Methods for method validation.

### 2.3. Qualitative Analysis by HPLC-QTOF-MS

Online HPLC-QTOF-MS analysis was made using an Agilent 1290 infinity LC system connected to a 6520 quadrupole time-of-flight mass spectrometer (Agilent Technologies, Santa Clara, CA, USA) equipped with an electrospray interface. The positive and negative ion ESI-MS experiments were conducted using conditions as follows: drying gas temperature, 320°C; drying gas (N_2_) flow rate, 10 l/min; nebulizer, 45 psi; capillary voltage, 4000 V for positive mode and 3500 V for negative mode; and fragmentor, 175 V. All the operation, acquisition, and analysis of data were made by Agilent Mass Hunter Acquisition Software Version B.04.00 (Agilent Technologies).

### 2.4. Experimental Animal Handling Procedure

Eight-week-old male Sprague-Dawley rats (250 ± 20 g) were purchased from the Promedican Pharmaceutical Co. Ltd. (animal license number: SCXK (hu) 2013-0016; Shanghai, China). Rats were housed in a well-ventilated room at constant room temperature (25 ± 2°C) and air humidity (50 ± 10%) with a light/dark cycle of 12 h. All animals were allowed ad libitum access to a standard diet (mouse crackers) and water throughout the study. The animals were acclimatized for 10 days prior to dosing treatment. All procedures for animal care and use were in accordance with the National Institute of Health (NIH) guidelines for the Care and Use of Laboratory Animals and approved by the Institutional Animal Care and Use Committee of China Pharmaceutical University (license number: SYXK (Su) 2016-0011). The rats were fasted for 12 h before the operation of the MCAO model but were allowed free access to water.

The MCAO operation using the intraluminal filament method was performed by one experienced researcher according to a previous method by Longa et al. [22] with some modifications, as previously described [10]. Briefly, animals were first anesthetized with chloral hydrate (3.5%, 350 mg/kg, i.p.). The right common carotid artery (CCA), the right external carotid artery (ECA), and the right internal carotid artery (ICA) were exposed and isolated from connective tissues. A poly-L-lysine-coated nylon monofilament (0.26 mm, 2636-A3, Beijing Cinontech Co. Ltd., Beijing, China) with a tip heat rounded diameter of 0.36 ± 0.02 mm was inserted into the ICA through the ECA until its tip is lodged in the anterior cerebral artery (ACA), a distance of 18 to 20 mm from the carotid bifurcation according to the weight of the animal, to obstruct the blood flow into the middle cerebral artery (MCA), thus achieving cerebral ischemia. After 2 h sustained ischemia, reperfusion was performed by the withdrawal of the inserted filament. Sham-operated rats received the same surgical procedures except that the arteries were not occluded. All surgical operations were done in a sterile environment. Rectal temperature of the animal was monitored by a thermistor and maintained at 37.0 ± 0.5°C with a thermostatically controlled heating pad (ALCBIO, Shanghai, People's Republic of China) during surgery and ischemia. The skull of rats was thinned using a drill at the skull surface of the core area supplied by the MCA (6 mm lateral and 2 mm posterior from the bregma) until a thin sheet of bone remains (observe the increasing redness), and the regional CBF was recorded by a laser Doppler flow meter (FLPI2, Moor Instruments Ltd., Axminster, UK).

In the preliminary experiments, the rats were subjected to 2 h MCAO followed by 1 d, 3 d, or 7 d of reperfusion. Samples from the sham rats were collected (NC); samples from the MCAO rats were collected at 1 d (1 d-M), 3 d (3 d-M), and 7 d (7 d-M) after reperfusion; samples from the HLJDD-treated group were collected at 1 d (1 d-HD), 3 d (3 d-HD), and 7 d (7 d-HD) after reperfusion; samples from the berberine-treated group were collected at 1 d (1 d-Ber), 3 d (3 d-Ber), and 7 d (7 d-Ber) after reperfusion; samples from the baicalin-treated group were collected at 1 d (1 d-Bai), 3 d (3 d-Bai), and 7 d (7 d-Bai) after reperfusion; and samples from the jasminoidin-treated group were collected at 1 d (1 d-Jas), 3 d (3 d-Jas), and 7 d (7 d-Jas) after reperfusion.

Serum and brain samples were subjected to analysis by the ^1^H NMR metabolomics approach. The binned NMR data were submitted to OPLS-DA. In the OPLS-DA score plots for serum and brain extracts (Supplementary Figures 1 and 2), the 1 d-M group is the furthest away from the NC group with 3d-M and 7d-M groups in between, suggesting that the most notable metabolic disturbance occurred at 1 d after reperfusion. To further investigate the metabolic perturbations induced by MCAO and HD/Ber/Bai/Jas, ^1^H NMR data of the M group was compared with that of NC and HD/Ber/Bai/Jas groups at each time point (1 d, 3 d, and 7 d after reperfusion) by OPLS-DA (Supplementary Figures S1 and 2), respectively. The results demonstrated that HLJDD-, berberine-, baicalin-, and jasminoidin-induced neuroprotections were effective on 1 d, 3 d, and 7 d after ischemia. We also detected the concentrations of berberine, baicalin, and jasminoidin in the blood and brain of rats at 15 min after regular administration and at 1 d, 3 d, and 7 d after reperfusion, by the HPLC-QTOF-MS/MS method. For details, see the Supplementary Information.

The pathologic processes caused by vascular injury after an occlusion of a cerebral artery are dynamic processes and can be separated into acute (hours), subacute (hours to days), and chronic (days to months). According to the results of NMR metabolomics in the preliminary experiment, the symptom of MCAO was the most severe at 1 d after reperfusion, so samples at 24 hour were further analyzed in our paper.

Rats were randomly assigned to six groups: (1) the sham operation group (NC, *n* = 30), (2) the MCAO model group (M, *n* = 50), (3) the HLJDD-treated group (HD, *n* = 40), (4) the berberine-treated group (Ber, *n* = 40), (5) the baicalin-treated group (Bai, *n* = 40), and (6) the jasminoidin-treated group (Jas, *n* = 40). The drugs were dissolved in 0.5% CMC-Na (carboxymethylcellulose sodium salt) and intragastrically (i.g.) administered to rats at dosages of 10 g/kg, 140 mg/kg, 66 mg/kg, and 55 mg/kg for HD-, Ber-, Bai-, and Jas-treated groups, with equal volume (10 ml/kg bodyweight), once a day for seven consecutive days. The sham and MCAO rats were administrated with equivalent volumes of 0.5% CMC-Na only.

### 2.5. Neurological Deficit Measurements

The neurological dysfunction of rats in the six groups was evaluated at 24 h after reperfusion as previously described [10]. The neurological scores were recorded by an investigator who was blinded to the experimental groups according to Longa et al.'s five-point scale: 0, no neurological deficit; 1, failure to extend to the right forelimb; 2, circling to the contralateral side; 3, falling to the contralateral side at rest; and 4, no spontaneous motor activity.

### 2.6. TTC Staining

Cerebral infarct volumes were measured by staining with 2,3,5-triphenyltetrazolium chloride (TTC, Sigma) to assess the severity of cerebral ischemia [23]. The brains were sectioned into six 2 mm thick coronal slices, stained with 2% TTC, incubated at room temperature (RT) for 30 minutes in the dark, and then fixed with 10% buffered formalin overnight. Normal tissue was stained rose red, and the infarct tissue was stained white. Slices stained with TTC were photographed and analyzed using image analysis software (Image-Pro Plus 6.0). Tests were conducted by an observer blinded to the treatment groups. To correct infarct volume (*V*_i_) for brain edema, the percentage of the infarction volumes (*I*%) was obtained using the following formula:

(1) $$\begin{array}{c} I\%=\frac{V_{c}-V_{i}}{V_{c}}\times 100\%. \end{array}$$

*V*_c_ = volume of the intact contralateral (left) hemisphere.

*V*_i_ = volume of intact regions of the ipsilateral (right) hemisphere [24].

### 2.7. Clinical Biochemistry, Histopathology, and Immunohistochemistry

The levels of oxidative stress-related biological components, including nitric oxide (NO), malondialdehyde (MDA), glutathione (GSH), glutathione disulphide (GSSG), Mn-superoxide dismutase (Mn-SOD), Cu/Zn-SOD, catalase (CAT), and glutathione peroxidase (GPx), were measured using commercially available kits (NanJing Jiancheng Bioengineering Institute).

The brain tissues were immersed in 10% neutral buffered formaldehyde for 24 h, embedded in paraffin, and sliced into 5 *μ*m thickness. The sliced sections were stained with hematoxylin-eosin (H&E) and examined by light microscopy (×400 magnification, Olympus DX45). The histopathology results were evaluated by Prof. Ning Su (Southeast University, Nanjing, China) who was blinded to the experiments.

For immunohistochemical examination, formalin-fixed, paraffin-embedded brain tissue sections were used and the activity of NF-*κ*B-p65 was evaluated by Goodbio Technology Co. Ltd. (Nanjing, China). The staining was photographed under light microscopy and analyzed using image analysis software (Image-Pro Plus 6.0).

### 2.8. Sample Preparation, ^1^H NMR Analysis, and Spectral Preprocessing

Details of serum and brain sample preparation, ^1^H NMR spectroscopic measurement, and spectral preprocessing are in the Supplementary Information.

### 2.9. Correlation Network Analysis

Metabolic correlation networks were performed using the R-package igraph software. The correlation networks could present the Pearson correlation coefficients among levels of metabolites and their structure similarity. Common Pearson correlation networks only visualized the correlations of metabolites in different status. However, such correlations were not causative relationships. In the networks, the solid lines between the molecules indicated a correlation between the molecules; the line colors of red and blue display positive and negative relationships, respectively. The nodes represented the metabolites, and the lines between the nodes indicated the biological relationships between the two correlation metabolites. Metabolites of similar structures were connected by the dotted lines indicating a possible biochemical reaction between the molecules. The addition of structure similarity information enriched the networks since that the substrates and products in nearly all the biochemical reactions should be similar. In this context, the high correlation between metabolites with great structure resemblance might reflect a theory of possible biochemical reaction between them and thus a causative effect.

### 2.10. Real-Time Quantitative RT-PCR Analysis

Real-time quantitative RT-PCR analysis was performed by using the primers listed in Supplementary Table S1, according to the method described in Supplementary Information.

### 2.11. Western Blot Analysis

Western blot assays were performed as described in Supplementary Information.

### 2.12. Statistical Analysis

Assays were conducted at least three times unless otherwise stated. All the experimental data, except for mortalities, were expressed as the means ± standard deviation (SD). Statistical significance was performed using Student's two-tailed *t*-test for comparison between two groups and one-way analysis of variance (ANOVA) followed by Tukey's multiple comparison test when the data involved three or more groups. A *p* value less than 0.05 was considered to be statistically significant.

## 3. Results

### 3.1. Quantitative and Qualitative Analyses

**Figure 1:**
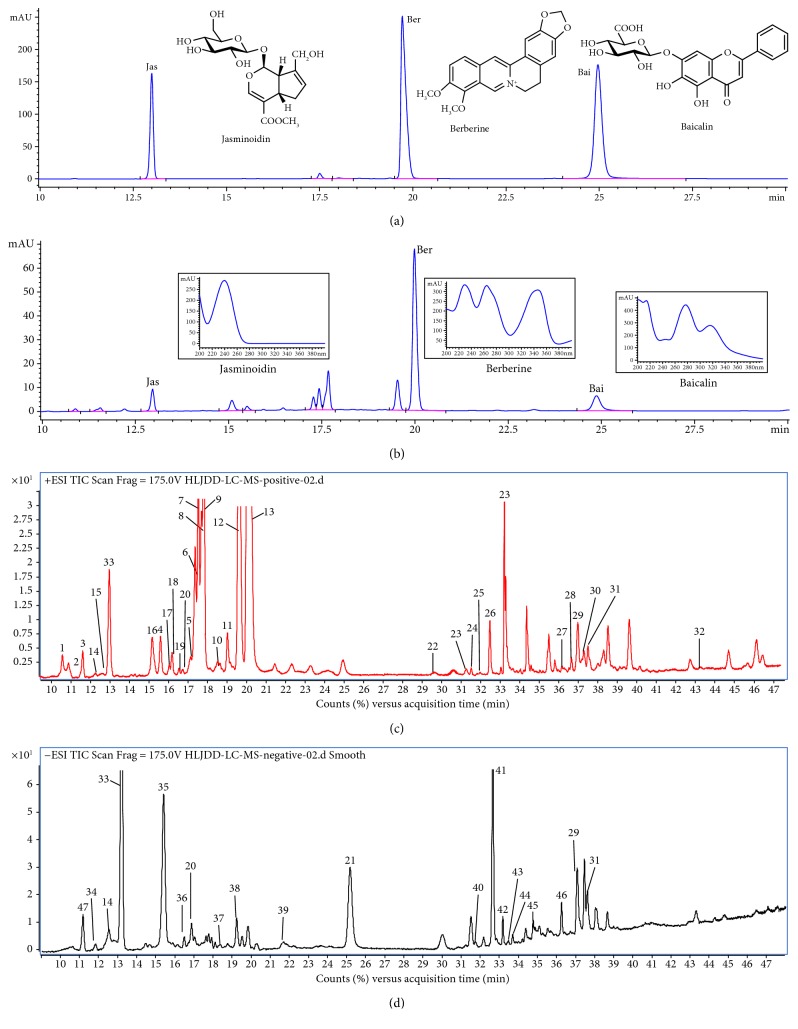
Profile of HPLC-UV and total ion current (TIC) chromatograms. (a) HPLC-UV chromatogram (10–30 min) of berberine, geniposide, and baicalin at 254 nm. (b) HPLC-UV chromatogram (10–30 min) of total extract of Huang-Lian-Jie-Du Decoction (HLJDD) at 254 nm. (c) Total ion current (TIC) chromatogram (10–48 min) of HLJDD extract analyzed by HPLC-QTOF-MS in a positive ion mode. Peaks 1–33 are listed in Supplementary Tables S2–4. (d) TIC chromatogram (10–48 min) of HLJDD analyzed by HPLC-QTOF-MS in a negative ion mode. Peaks 34–47 are listed in Supplementary Tables S5 and 6.

The optimized HPLC method was subsequently applied to the simultaneous quantitative analysis of berberine, baicalin, and jasminoidin in HLJDD extract (Figures 1(a) and 1(b) and Supplementary Figure S3), and their contents were determined to be 5.32%, 2.51%, and 2.09%, respectively. As shown in Figures 1(c) and 1(d), 47 components in HLJDD extracts were identified by HPLC-QTOF-MS and listed in Supplementary Tables S2–6, mainly including alkaloids, iridoids, and flavonoids.

### 3.2. Neurological Deficit, Ischemic Infarct, and Histopathological Assessments

**Figure 2:**
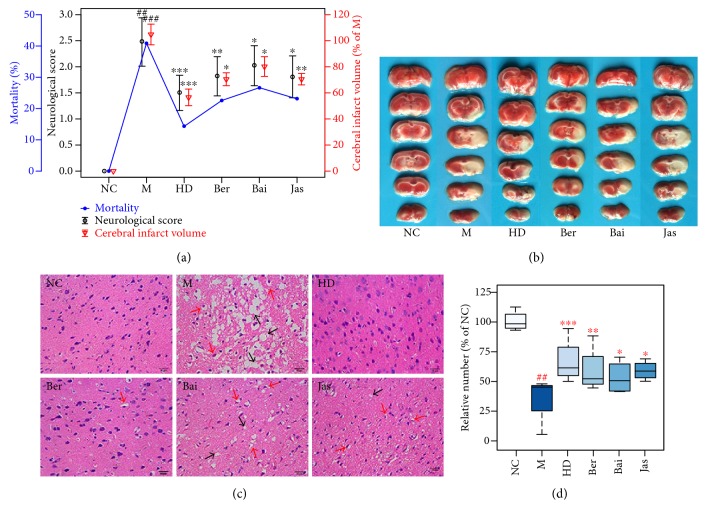
Neurological deficit, ischemic infarct, and histopathological assessment. (a) Mortality, infarct volume examinations, and neurobehavioral scores. (b) TTC sting of brains (*n* = 6). (c) Histopathological examination of brain tissues by hematoxylin-eosin (H&E) staining (×400 magnification; *n* = 4): neuronal loss and presence of numerous vacuolated spaces (black arrow) and disordered neuron arrangement (red arrow). (d) The abnormal neurons were counted and expressed relatively to the sham group (*n* = 4 in each group). ^##^*p* < 0.01 and ^###^*p* < 0.001, MCAO group versus sham group; ^∗^*p* < 0.05, ^∗∗^*p* < 0.01, and ^∗∗∗^*p* < 0.001, drug-treated groups versus MCAO group.

**Figure 3:**
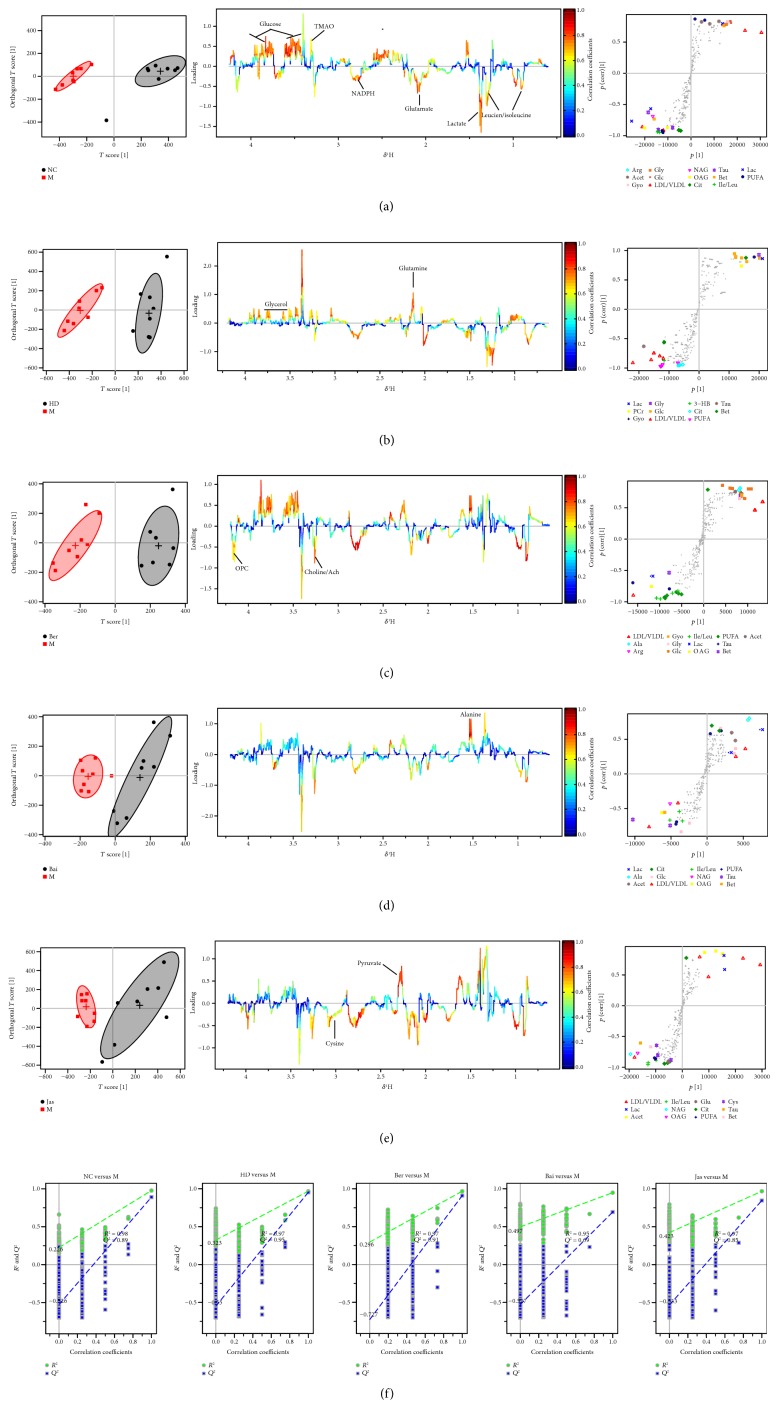
OPLS-DA based on ^1^H NMR data from serum of all groups. OPLS-DA of serum ^1^H NMR data of NC, M, HD, Ber, Bai, and Jas groups after the removal of water signals: score plots, the corresponding loading plots and S-plots ((a) NC versus M, (b) HD versus M, (c) Ber versus M, (d) Bai versus M, and (e) Jas versus M). OPLS-DA scatter plot from serum (f) of the statistical validations obtained by 2000 times permutation tests, with *R*^2^ and *Q*^2^ values in the vertical axis and the correlation coefficients (between the permuted and true class) in the horizontal axis, and OLS line representing the regression of *R*^2^ and *Q*^2^ on the correlation coefficients.

The middle cerebral artery blood flow in all groups had decreased 20–30% of preischemia levels immediately after the occlusion was used to confirm that (Supplementary Figure S4), indicating excellent models. At 24 h after reperfusion, the mortality, infarct volumes, functional response, and morphological abnormality of MCAO rats treated with HD, Ber, Bai, and Jas were assessed in this study. The results showed that (1) the mortalities and scores of the HD and Ber treatment groups were significantly lower than those of the model group (Figure 2(a)); (2) In HD-, Ber-, and Jas- treated rats, the percentage of infarct volume was significantly reduced compared to that of model control rats (Figure 2(b)); (3) HD and Ber treatments remarkably ameliorated pathological changes in the brain tissue of MCAO rats, while pathological abnormalities were occasionally observed for the Bai and Jas groups (Figures 2(c) and 2(d)).

### 3.3. Metabolite Identification in ^1^H NMR Spectra of Serum and Tissues

Representative 500 MHz ^1^H NMR spectra of serum and brain samples are shown in Supplementary Figure S5 with the metabolites labelled. Twenty-six metabolites in serum and forty-two metabolites in brain extracts were identified by querying publicly accessible metabolomics databases such as Madison (http://mmcd.nmrfam.wisc.edu/) and HMDB (http://www.hmdb.ca/), aided by Chenomx NMR Suite (Version 8.1, Chenomx Inc., Edmonton, Canada). Detailed information about the metabolites is listed in Supplementary Tables S7 and 8.

### 3.4. Multivariate and Univariate Analyses of ^1^H NMR Spectral Data of All Groups

**Figure 4:**
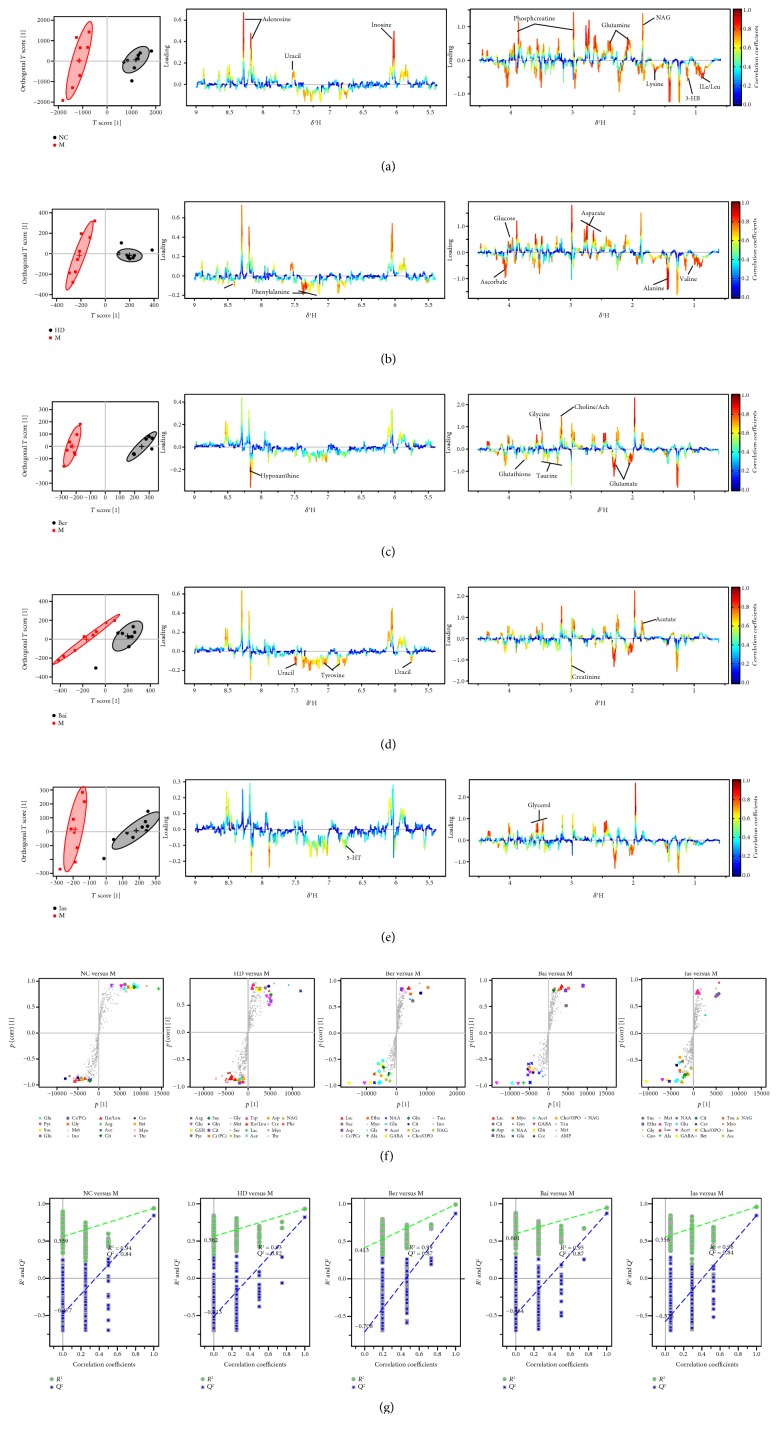
OPLS-DA based on ^1^H NMR data from brain extracts of all groups. OPLS-DA score plots and the corresponding color-coded loading plots ((a) NC versus M, (b) HD versus M, (c) Ber versus M, (d) Bai versus M, and (e) Jas versus M) and S-plots (f) (*n* = 8). OPLS-DA scatter plots from brain extracts (g) of the statistical validations obtained by 2000 times permutation tests, with *R*^2^ and *Q*^2^ values in the vertical axis and the correlation coefficients (between the permuted and true class) in the horizontal axis, and OLS line representing the regression of *R*^2^ and *Q*^2^ on the correlation coefficients.

The whole ^1^H NMR data of serum and brain extracts were analyzed by orthogonal partial least squares discriminant analysis (OPLS-DA) to explore the effects of HD, Ber, Bai, and Jas on MCAO rats. To investigate the metabolic perturbations induced by MCAO and HD/Ber/Bai/Jas, ^1^H NMR data belonging to the MCAO group (M) were compared with those belonging to the sham group (NC) and HD/Ber/Bai/Jas group by OPLS-DA (Figures 3 and 4), respectively. In the score plots, the showcased clusters corresponded to metabolic patterns in different groups with each point representing one sample. In the OPLS-DA score plots of the serum and brain extracts, NC, HD, Ber, Bai, and Jas groups were completely separated from the M group, demonstrating severe metabolic disturbance induced by MCAO, which could be ameliorated after HD, Ber, Bai, and Jas treatments. OPLS-DA loading plots and S-plots were generated to identify the metabolites responsible for the differentiation in the score plots. The color-coded loading plots were color-encoded according to the absolute correlation coefficient of each variable to grouping; a hot-colored signal (red) indicated more significant contribution to class separation than a cold-colored one (blue) and is presented in a covariance-based pseudospectrum. S-plots were another way to identify significantly altered metabolites, which should be located in the upper right or lower left quadrant and farther away from the origin.

These important differential metabolites identified by OPLS-DA loading plots and S-plots were further tested for their between-groups difference using univariate analysis. The fold change values of metabolites in the sham-operated or drug-treated rats relative to the MCAO group and the corresponding *p* values adjusted by the Benjamini-Hochberg method [25] were calculated and visualized by fold change plots (Supplementary Figure S6); for details, see the Supplementary Information.

### 3.5. SUS Shared and Unique Structure Analysis

**Figure 5:**
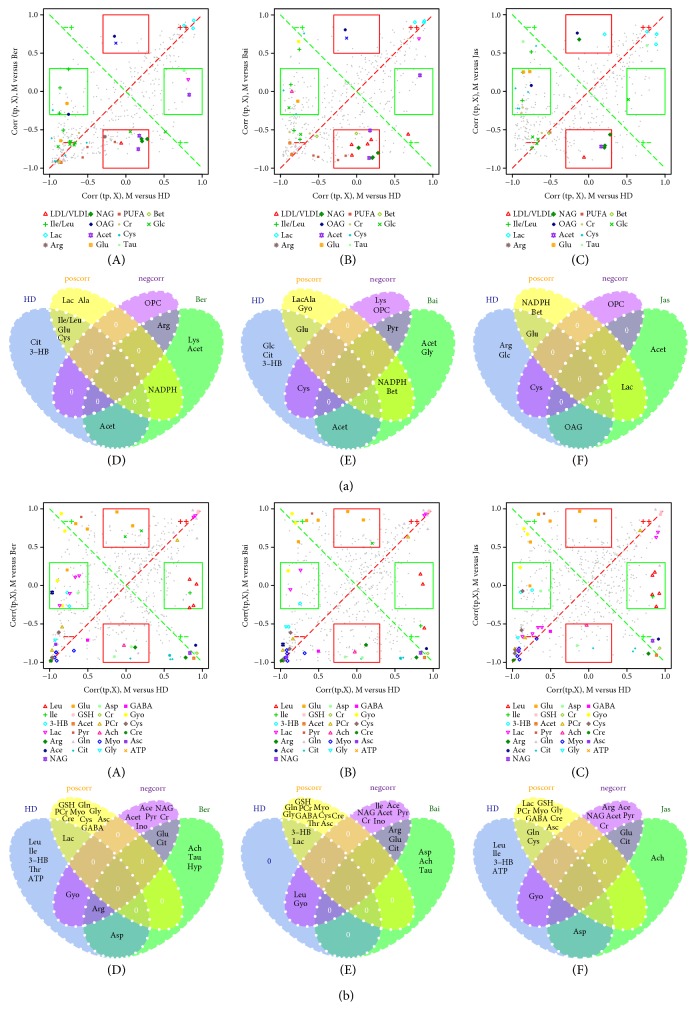
SUS-plot between HD and Ber (A), HD and Bai (B), and HD and Jas (C) of metabolites from serum (a) and the brain extracts (b). Metabolites (VIP > 1) in unique effects were found close to either the *x*- or *y*-axis for HD (red rectangle) and Ber/Bai/Jas (green rectangle), while the shared effects were located on the diagonals (metabolites close to the red diagonal line are positive correlation and those close to the green diagonal line are negative correlation). The shared and unique structures of metabolites were also visualized in Venn plots between HD and Ber (D), HD and Bai (E), and HD and Jas (F).

In the present study, an extension of the S-plot, the SUS-plot (shared and unique structure), was applied to further compare the outcome of two treated groups (e.g., HD versus Ber, HD versus Bai, and HD versus Jas) to that of the MCAO group. The SUS-plot combines the corr (tp, X) profiles from two models, in one 2D plot. As the SUS-plot displays correlation, it should be scaled between −1 and +1 for both axes. Metabolites close to the diagonal will be shared between classes, and metabolites outside the diagonal will be unique for the specified class; see results in Figures 5(a) A–C and 5(b) A–C. Then, the four regions of the SUS-plot were improvably visualized in Venn plots (Figures 5(a) D–F and 5(b) D–F).

As shown in Figure 5, the shared and unique structures of metabolites from brain extracts were found in the HD group compared to Ber/Bai/Jas groups, based on OPLS-DA SUS-plots. Glu, Ach, and Cit were the unique metabolites for all Ber, Bai, and Jas groups; ATP, Gyo, and 3-HB were the unique metabolites of HD; and Acet, Cr, Ace, and Pyr were the negative correlation between HD and Ber/Bai/Jas, suggesting that HD did better in ameliorating the disordered energy metabolism in MCAO rats than Ber and Jas.

### 3.6. Correlation Network of Differential Metabolites

**Figure 6:**
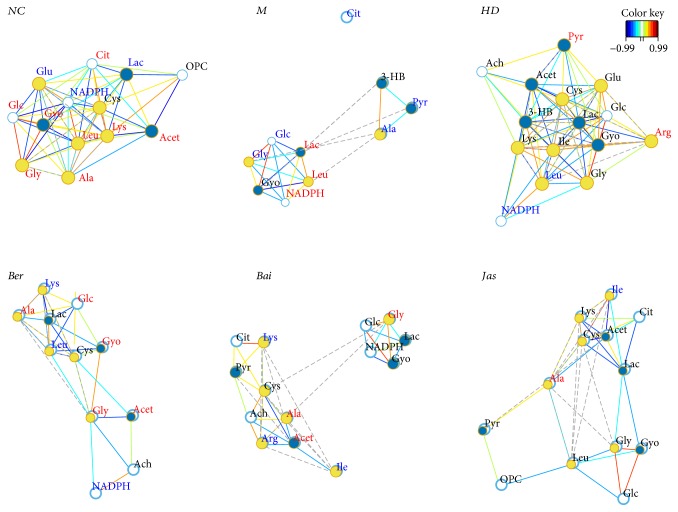
Correlation network of differential metabolites in serum of all groups. Only correlations with absolute values of correlation coefficients greater than 0.65 and *p* values <0.05 were kept. The nodes represent the metabolites, and the lines between the nodes indicate the biological relationships between the two corresponding metabolites. The red (blue) metabolites represent the upregulated metabolites (the downregulated metabolites) in MCAO rats compared with the sham rats or in drug-treated rats compared with the MCAO rats. The solid lines between the molecules indicate a correlation between the molecules; the line colors of red and blue display positive and negative relationships, respectively. Metabolites of similar structures were connected by the dotted lines, indicating a possible biochemical reaction between the molecules.

Instead of only investigating the individual metabolite variation, metabolic correlation networks were performed using the R-package igraph software to decipher the biological correlation between the candidate biomarkers. As shown in Figure 6, the networks were constructed based on the differential metabolites in the serum of the sham, MCAO, and drug-treated rats selected based on OPLS-DA loadings/S-plots. For the sham network, citrate, the key intermediate of the TCA cycle, was highly correlated with many metabolites, including glucose, leucine, acetoacetate, glutamate, *β*-nicotinamide adenine dinucleotide phosphate (NADPH), cysteine, lactate, and phosphorylcholine (OPC). However, these correlations were absent for MCAO rats. For MCAO rats, glycolysis-related metabolite lactate was located in the center of the network with an elevated tendency. A strong negative correlation was observed between glucose and lactate, indicating a shift of energy production from aerobic respiration toward anaerobic glycolysis. Strong correlations among 3-hydroxybutyrate (3-HB), pyruvate, and alanine also suggested that the metabolism of ketone bodies was activated to replenish energy. The perturbed correlation network in the MCAO rats could be partially rectified by HLJDD and its three principals.

**Figure 7:**
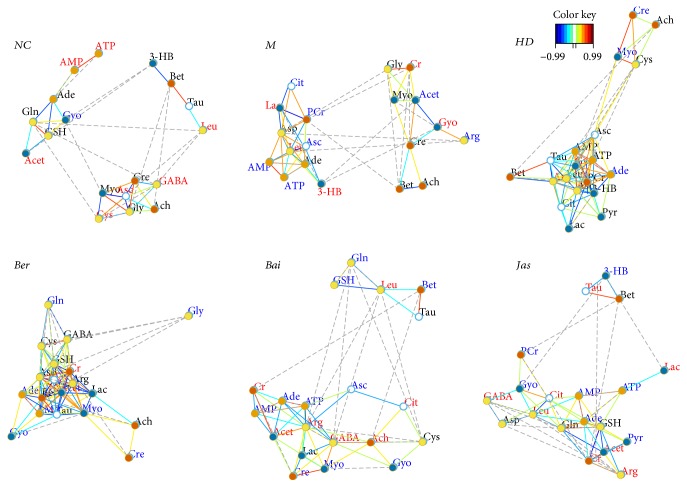
Correlation network of differential metabolites in brain extracts of all groups. Only correlations with absolute values of correlation coefficients greater than 0.85 and *p* values <0.05 were kept. The nodes represent the metabolites, and the lines between the nodes indicate the biological relationships between the two corresponding metabolites. The red (blue) metabolites represent the upregulated metabolites (the downregulated metabolites) in MCAO rats compared with the sham rats or in drug-treated rats compared with the MCAO rats. The solid lines between the molecules indicate a correlation between the molecules; the line colors of red and blue display positive and negative relationships, respectively. Metabolites of similar structures were connected by the dotted lines, indicating a possible biochemical reaction between the molecules.

Some clear differences were observed between the sham and ischemia/reperfusion (I/R) rats. In brains (Figure 7), high negative correlations were observed between lactate and citrate and ascorbate and phosphocreatine (PCr) in MCAO rats, indicating also a shift of energy production means toward anaerobic glycolysis. Ketone bodies (e.g., 3-HB and acetoacetate) appeared highly correlated with many metabolites. A positive correlation was observed between the levels of creatine (Cr) and PCr, showcasing the accelerated utilization of the Cr-PCr system and ketone bodies for ATP production in ischemic stroke. Strong correlations between GSH and glutamate were observed in the sham rats, which were absent in MCAO rats. The same was found for the betaine-taurine pair, which was absent in MCAO rats, and presented in sham rats and treatment groups. Betaine and taurine functioned as important organic osmolytes in brains, thus directly related to osmotic equilibrium. The absent of betaine-taurine correlation in MCAO rats suggested the disturbed ionic balance by I/R, which eventually resulted in brain edema.

Correlation network analysis provides evidence of energy imbalance and oxidative stress in ischemia reperfusion injury, such as glycolysis-derived metabolites, TCA cycle intermediates, phosphate compounds, ketone bodies, and glutathione metabolism, concerning the treatment of the three principals from HLJDD in ischemic stroke.

### 3.7. Effects of HLJDD and Its Three Principals on Oxidative Stress

**Figure 8:**
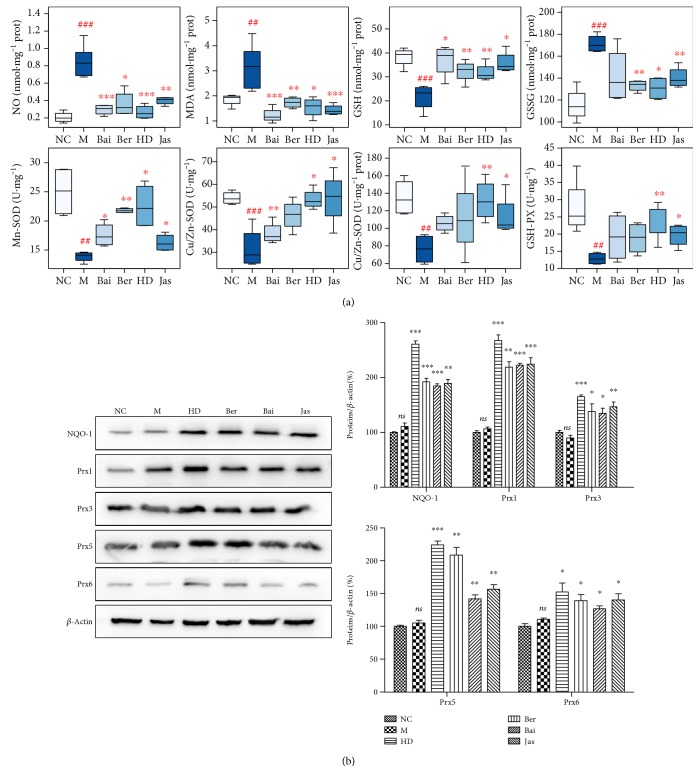
Effects of HLJDD and its three principal components on oxidative stress. (a) Boxplots for brain tissue levels of NO, MDA, CAT, Mn-SOD, Cu/Zn-SOD, GSH, GSSG, and GPx in each group (*n* = 6). The lines drawn at the bottom of each box, in the box, and at the top of the box represented the 1st, 2nd, and 3rd quartiles, respectively. The whiskers extended to ±1.5 times the interquartile range (from the 1st to 3rd quartile). All the data obtained were expressed as mean ± standard deviation (SD) (*n* = 6). ^##^*p* < 0.01 and ^###^*p* < 0.001, MCAO group versus sham group; ^∗^*p* < 0.05, ^∗∗^*p* < 0.01, and ^∗∗∗^*p* < 0.001, drug-treated groups versus MCAO group. (b) The protein level of NQO-1, Prx1, Prx3, Prx5, and Prx6 in brains treated with HLJDD and its three principals (*n* = 6). Results of quantitative analysis values are expressed as mean ± SD (*n* = 6). MCAO group versus sham group; ^∗^*p* < 0.05, ^∗∗^*p* < 0.01, and ^∗∗∗^*p* < 0.001, drug-treated groups versus MCAO group. ns, no significant difference.

In our experiment, the levels of oxidative stress-related biologicals were measured (Figure 8(a)). Compared with the sham group, NO and MDA, which are the oxidative stress markers, were significantly increased in the I/R group and were decreased in the HD, Ber, and Jas groups. The activities of the antioxidases Mn-SOD, Cu/Zn-SOD, CAT, and GPx were apparently inhibited in the M group compared with the sham group, which were greatly augmented by the treatment with HD, Ber, and Jas. I/R produced a notable reduction in the quantity of GSH and correspondingly a significant accumulation of GSSG in the M group, which was reversed by HD, Ber, and Jas treatments.

In addition, the expressions of peroxidases (Prx1, Prx3, Prx5, and Prx6) and NAD(P)H:quinone oxidoreductase (NQO-1) were determined by Western blot analysis (Figure 8(b)). The results revealed that rats in the MCAO group had a slight increase in the protein expressions of Prx1, Prx3, Prx5, Prx6, and NQO-1. As compared with the MCAO group, HD, Ber, and Jas significantly increased the expression levels of these proteins.

### 3.8. Effects of HLJDD and Its Three Principals on Autophagy

**Figure 9:**
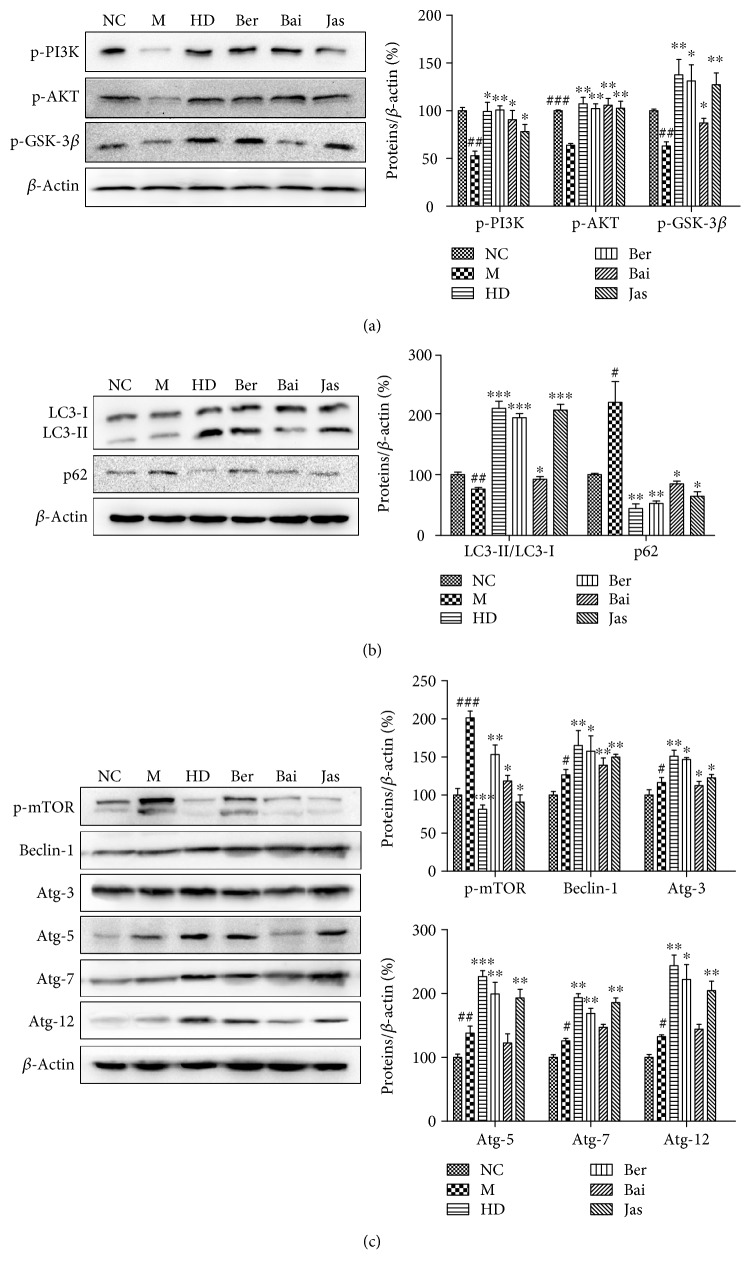
Effects of HLJDD and its three principal components on autophagy. (a) The protein levels of p-PI3K, p-AKT, and p-GSK-3*β* in brains treated with HLJDD and its three principals (*n* = 6). (b) The protein levels of LC3-I, LC3-II, and p62 in brains treated with HLJDD and its three principals (*n* = 6). (c) The protein levels of p-mTOR, beclin1, Atg-3, Atg-5, Atg-7, and Atg-12 in brains treated with HLJDD and its three principals (*n* = 6). Results of quantitative analysis values are expressed as mean ± SD (*n* = 6). ^#^*p* < 0.05, ^##^*p* < 0.01, and ^###^*p* < 0.001, MCAO group versus sham group; ^∗^*p* < 0.05, ^∗∗^*p* < 0.01, and ^∗∗∗^*p* < 0.001, drug-treated groups versus MCAO group.

Compared with the MCAO group, the greatly increased ratio of microtubule-associated protein light chain 3- (LC3-) II/LC3-I and the decreased expression of the polyubiquitin-binding protein p62 were observed in the HD, Ber, Bai, and Jas groups (Figure 8(c)), manifesting an induction of autophagy by the four treatments. To confirm the inference, the expressions of several autophagy-related proteins, such as phospho-mammalian target of rapamycin (p-mTOR), beclin-1; Atg-3, Atg-5, Atg-7, and Atg-12; phopho-phosphatidylinositol-3 kinases (p-PI3K), phospho-protein kinase B (p-AKT), and phospho-glycogen synthase kinase-3 beta (p-GSK-3*β*), were analyzed using Western blot analysis (Figure 9). Compared with those in the MCAO group, in addition to p-mTOR, all of these proteins were markedly increased in all four treatment groups, demonstrating the ability of HLJDD and its three principals to induce autophagy.

### 3.9. Effects of HLJDD and Its Three Principals on Inflammatory Responses

**Figure 10:**
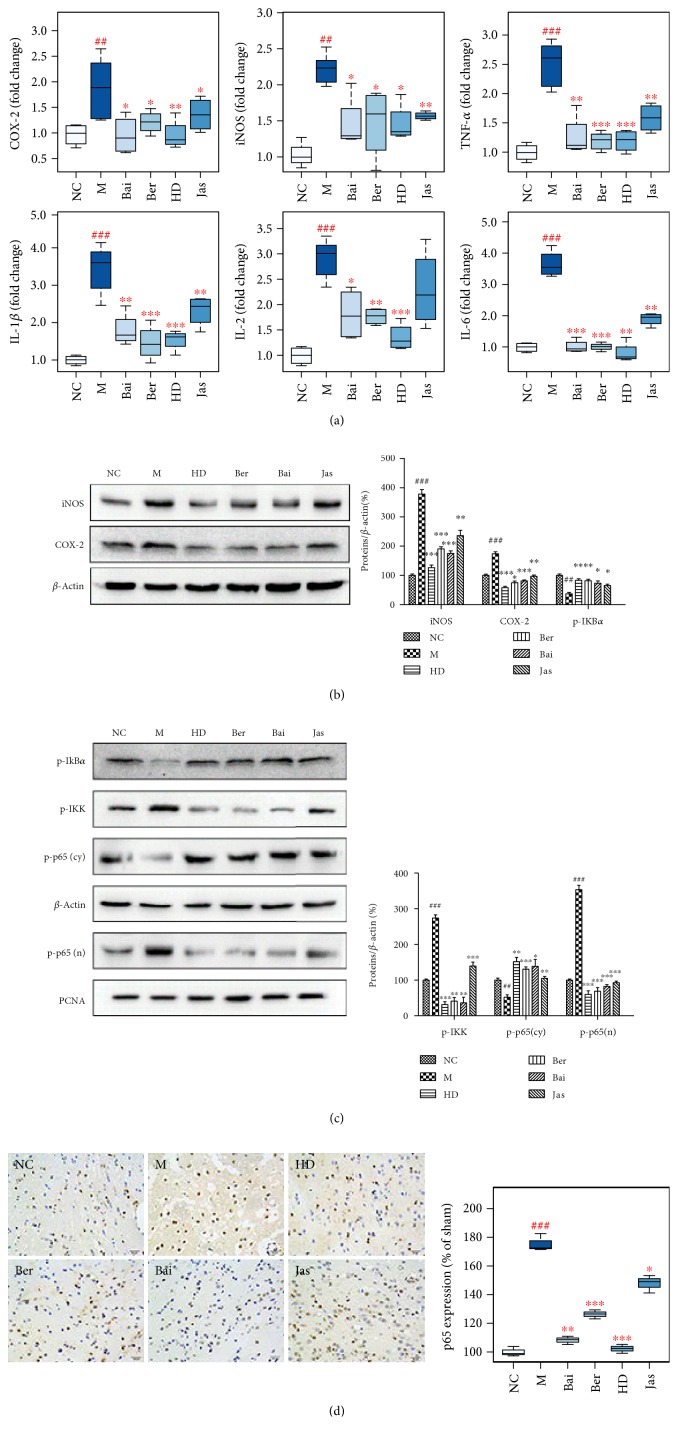
Effects of HLJDD and its three principal components on inflammatory responses. (a) Boxplots for the gene expression levels of COX2, iNOS, TNF-*α*, IL-1*β*, IL-2, and IL-6 in brains by qRT-PCR in each group (*n* = 6). The line drawn at the bottom of each box, in the box, and at the top of the box represented the 1st, 2nd, and 3rd quartiles, respectively. The whiskers extended to ±1.5 times the interquartile range (from the 1st to 3rd quartile). All the data obtained were expressed as mean ± standard deviation (SD). ^##^*p* < 0.01 and ^###^*p* < 0.001, MCAO group versus sham group; ^∗^*p* < 0.05, ^∗∗^*p* < 0.01, and ^∗∗∗^*p* < 0.001, drug-treated groups versus MCAO group. (b) The protein level of COX-2 and iNOS in brains treated with HLJDD and its three principals (*n* = 6). (c) The protein level of cytosolic p-IKB*α*, p-IKK, and p-p65 and nuclear p-p65 in brains treated with HLJDD and its three principals (*n* = 6). (d) The protein level of p65 in brain tissues of rats treated with HD, Ber, Bai, and Jas determined by immunohistochemical staining. Results of quantitative analysis values are expressed as mean ± SD (*n* = 5). ^##^*p* < 0.01 and ^###^*p* < 0.001, MCAO group versus sham group; ^∗^*p* < 0.05, ^∗∗^*p* < 0.01, and ^∗∗∗^*p* < 0.001, drug-treated groups versus MCAO group.

In our experiment, the gene and protein expressions of inducible nitric oxide synthase (iNOS) and cyclooxygenase-2 (COX-2) were significantly increased in the MCAO rats (Figure 10), demonstrating a strong inflammatory response. In addition to these proinflammatory enzymes, proinflammatory cytokines such as tumor necrosis factor-*α* (TNF-*α*), interleukin-1*β* (IL-1*β*), interleukin-2 (IL-2), and interleukin-6 (IL-6) were also increased at the mRNA level after I/R. HD, Ber, and Bai treatments effectively suppressed the activation of I/R-induced proinflammatory enzymes (COX-2 and iNOS) and proinflammatory cytokines (e.g., TNF-*α*, IL-1*β*, IL-2, and IL-6).

The effects of HLJDD and its three principals on the expression of phospho-NF-*κ*B- (nuclear factor-kappa B-) p65 (p-p65) after I/R were investigated using immunohistochemical analysis and Western blotting. The protein expressions of nuclear p-p65 and cytoplasmic phospho-I-kappa-B kinase (p-IKK) were significantly increased in the MCAO group, which were dramatically decreased by HD, Ber, and Bai and slightly decreased by Jas. Correspondingly, the expression of the p-p65 inhibitor phospho-inhibitor of NF-kappa B*α* (p-IKB*α*) in the cytosolic fraction was significantly decreased in the MCAO group, which could be markedly increased by HD, Ber, and Bai treatments.

## 4. Discussion

In this study, complemented with mortality, neurologic deficit scoring, infarct volume, histopathological inspection, clinical biochemistry, immunohistochemical assay, qRT-PCR and Western blot analysis, a ^1^H NMR-based metabolomics approach was adopted to explore the role of the three major active components of HLJDD, namely, berberine, baicalin, and jasminoidin on the treatment of ischemic stroke and to explore the underlying mechanisms. Correlation network analysis of the metabolic variations indicated that the most significantly affected pathways in MCAO rats were those involved in energy metabolism and oxidative stress.

### 4.1. Energy Metabolism

Cerebral I/R injury was caused by the disruption and then subsequent restoration of glucose and oxygen supply to the brain. When glucose and oxygen supply is limited, ATP synthesis by aerobic metabolism of glucose through electron transport chain- (ETC-) mediated oxidative phosphorylation is inhibited, which was shown by markedly decreased levels of glucose, ATP, and pyruvate in MCAO rats. Furthermore, little pyruvate can enter the tricarboxylic acid (TCA) cycle under these conditions; therefore, citrate also decreases. The lactate (byproduct of anaerobic respiration) level was significantly increased, suggesting a shift toward the much less efficient anaerobic glycolytic ATP synthesis, which is inadequate to support normal cerebral function. This shift results in an increase in other means to produce energy, such as the Cr-PCr system and ketone bodies. The Cr-PCr system, through the creatine kinase (CK) reaction, plays a crucial role in maintaining a constant ATP level [26]. Their decreased levels in the brain suggested an accelerated conversion to ATP. Ketone bodies, for example, 3-HB and acetoacetate, may also serve as fuel if the brain is starving. The ketone bodies were transferred from the serum to the brain to replenish an insufficient energy supply [27], which was supported by their observed decrease and increase, in serum and brains of MCAO rats, respectively.

The results indicate that energy metabolism was severely damaged in the MCAO rats. HLJDD and its three principals greatly improved the damaged energy metabolism in MCAO rats as evidenced by their ability to increase the glucose levels, thus increasing energy availability. As the major pathway in energy production, the TCA cycle was enhanced by HLJDD and its three components as indicated by the marked increase in citrate and pyruvate. With the improved energy supply, the other energy production means were no longer necessary as exemplified by the elevated Cr and PCr levels and reduced ketone body levels in brains of treatment rats as compared with those in the MCAO rats.

### 4.2. Oxidative Stress

Reperfusion after ischemia brought oxygen and glucose to neurons in an amount exceeding their normal consumption, which would trigger reperfusion injury. Reperfusion injury in brain tissues is well documented with oxidative stress as the most notable feature [28]. Oxidative stress is an imbalance between the production of free radicals, in particular, reactive oxygen species (ROS), and the ability of the organism to remove them, which is a major mechanism underlying cerebral ischemia-reperfusion injury [29, 30]. The brain is very susceptible to oxidative stress-induced damage due to its rapid oxidative metabolic activity, high content of polyunsaturated fatty acids, relatively low antioxidant capacity, and inadequate neuronal cell repair ability [31]. Cerebral ischemia is known to induce the generation of ROS, deemed as one of the earliest signs of tissue injury after reperfusion of the ischemic organ [32].

The significantly increased levels of NO (the precursor of peroxynitrite) and MDA (the product of lipid peroxidation) in the MCAO group indicated a severe oxidative damage induced by ischemia/reperfusion [33]. The body also developed defense mechanisms against oxidative damage. NQO-1 was increased in the model group, which was an important cytoprotective gene. Antioxidant enzymes, such as SOD, CAT and GPX, and low-molecular weight ROS scavengers, such as GSH and ascorbate, are critical to attenuate the injury induced by ischemia/reperfusion. Their activities were severely decreased in the brain of MCAO rats (compared with the sham rats). SOD and CAT are metalloproteins that catalyze “dismutation” reactions, which detoxify O_2_^−^ and H_2_O_2_, respectively. SOD is the most studied antioxidant enzyme in stroke. There are two major endogenous isoforms of intracellular superoxide SOD: Cu/Zn-SOD is principally found in the cytosol and lysosomal fractions and manganese SOD (Mn-SOD) is primarily found in the mitochondrial matrix. Compared with the sham rats, Cu/Zn-SOD and Mn-SOD in the MCAO brain were severely decreased.

Under the catalysis of GPx, GSH is oxidized to disulfide (GSSG) [34]. The GSSG is converted back to GSH by glutathione reductase (GR), which reduces equivalents derived from NADPH. GPx and GSH in MCAO brains were severely decreased compared with those in the sham-operated rat brains.

Peroxiredoxins (Prxs) work in tandem with GPx to maintain constant cellular peroxide levels. Prxs have been found to be induced in response to oxidative stress [35] and have shown protection against oxidative stress when overexpressed in animals, thus playing a protective role in cerebral ischemia-reperfusion injury [36]. In our study, cerebral ischemia slightly increased the expression of Prx isozymes (Prx1, Prx3, Prx5, and Prx6).

Compared with the I/R group, HD, Ber, and Jas lowered the increased levels of NO and MDA and enhanced the activities of antioxidases (SODs, CAT, and GPx) and peroxiredoxins (Prxs) and the levels of NQO-1 and GSH. These biochemical parameters combined with the results of Western blotting demonstrated the protective ability of HD, Ber, and Jas to counteract ROS and ameliorate the status of oxidative stress induced by I/R injury.

### 4.3. Autophagy

Autophagy, an important cell clearance system for degrading wastes or impaired organelles into micromolecules for reuse, has been reported to play a protective role in I/R [37, 38]. LC3 and p62 are important proteins in autophagy, during which cytoplasmic pattern LC3 (LC3-I) is converted to autophagosomal membrane LC3 (LC3-II), leading to increased LC3-II or LC3-II/LC3-I levels [39]. However, p62 expression level is generally decreased during autophagy [40].

Our previous study suggested that the AKT signal transduction pathway may be involved in the protection of HLJDD against cerebral ischemia injury [41]. AKT is known to function by phosphorylation and subsequent inactivation of many proteins, with serine/threonine kinase GSK-3*β* as one of its primary targets [42]. The AKT/GSK-3*β* signaling pathway played an important role in the regulation of autophagy [43].

In this study, we demonstrate that HD, Ber, and Jas elevated the LC3-II/LC3-I, beclin-1, Atg-3, Atg-5, Atg-7, and Atg-12 levels, decreased the expression of p62 and restrained the activation of mTOR (one of the most important proteins to negatively regulate autophagy), and upregulated the phosphorylation of AKT and its related substrate GSK-3*β* at 24 h of reperfusion. These findings suggest that Ber and Jas promote autophagy by increasing LC3-II/LC3-I, beclin-1, and Atg expression, activating the AKT/GSK3*β* signaling pathway, and by decreasing p62 expression, restraining the phosphorylation of mTOR.

### 4.4. Inflammation

NF-*κ*B, a redox-sensitive transcription factor regulating a battery of inflammatory genes, played an essential role in the regulation of postischemic inflammation, which is against the recovery from an ischemic stroke [44]. When unstimulated, NF-*κ*B is located in the cytoplasm as an inactive heterodimer composed of two subunits, p50 and p65. The heterodimer forms a complex with the inhibitory protein I*κ*B-*α*, retaining the protein in the cytoplasm. ROS can activate the enzyme I*κ*B kinase (IKK), which in turn induces I*κ*B-*α* phosphorylation, followed by I*κ*B-*α* degradation and NF-*κ*B p65/p50 activation. The activated NF-*κ*B is then translocated into the nucleus where the protein binds to specific sequences of DNA called response elements (RE), which finally leads to the expression of multiple proinflammatory cytokines, such as TNF-*α*, IL-1*β*, IL-2, and IL-6, and inducible proinflammatory enzymes (COX-2 and iNOS), exacerbating the inflammatory progression of brain damages [45, 46].

In the present study, iNOS and COX-2 were upregulated at 24 h after reperfusion. Similarly, proinflammatory genes, such as TNF-*α*, IL-1*β*, IL-2, and IL-6, were present in low amounts in the brain of sham-operated rats and were induced by I/R. The upregulation of iNOS was paralleled by an increased production of NO, which may react with ROS to produce peroxynitrites, notorious in the process of neuronal damage triggered by cerebral I/R [47].

HD, Ber, and Bai treatments protected the rat brain from I/R-induced nuclear translocation of NF-*κ*B-p65 and overexpression of proinflammatory genes (TNF-*α*, IL-1*β*, IL-2, and IL-6) and enzyme (iNOS, COX-2). HD, Ber, and Bai could inhibit kinases of I*κ*B and IKK by interfering with their phosphorylation or ubiquitination, preventing the translocation of NF-*κ*B into the nucleus and the transcription of proinflammatory cytokines.

HD, Ber, and Bai treatments significantly enhanced the phosphorylation of PI3K and AKT proteins. PI3K/Akt and NF-*κ*B signaling pathways may be functionally interconnected and not acting independently. Activation of the PI3K/Akt pathway can exert its prominent neuroprotective effect against cerebral I/R injury by regulation of inflammation [48, 49]. AKT pathway activation has been shown to abolish NF-*κ*B-driven activation of gene expression [50] and has been suggested to contribute to the inhibition of NF-*κ*B activation by modulating the transactivation capacity of the NF-*κ*B p65 subunit [51]. Therefore, HD, Ber, and Bai might affect NF-*κ*B activation via PI3K/Akt signaling cascade. Direct experimental evidences should be incorporated to determine whether the activation of PI3K/Akt and inhibition of NF-*κ*B activity by HD, Ber, and Bai represent a direct mechanism underlying the anti-inflammation of HD, Ber, and Bai during transient cerebral I/R.

## 5. Conclusions

**Figure 11:**
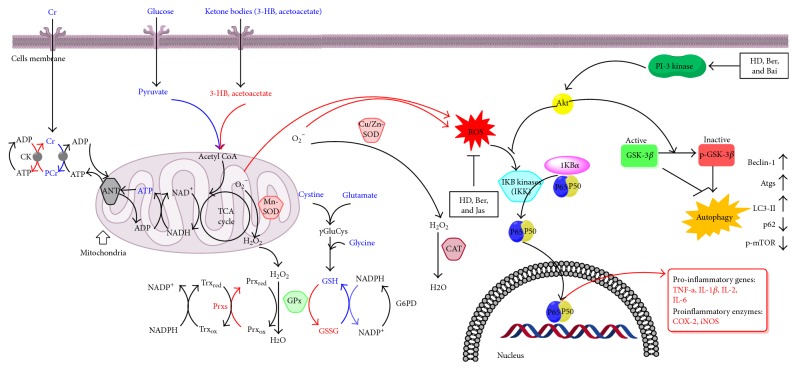
The signaling pathway changes triggered by HLJDD and its three principals. Red arrow represents the increased metabolites in MCAO rats and blue arrow represents the decreased metabolites in MCAO rats, as determined by ^1^H NMR. HLJDD and its three principals greatly improved the damaged energy metabolism and significantly increased cellular antioxidants to scavenge overgenerated ROS during I/R. Autophagy could be induced by the inactivation of GSK-3*β*, which in turn was regulated by Akt. Upon the treatment of HD, Ber, and Jas, GSK-3*β* was inactivated via the regulation of the Akt, a positive regulator of p-GSK-3*β* (arrow: positive regulation; blunt-ended line: negative regulation). Similarly, the level of p-GSK-3*β* was increased by HD, Ber, and Jas. HD, Ber, and Bai treatments inactivated p65 by regulation of IKK or PI3K/Akt. COX2 and iNOS are downstream of the p65-active signaling pathway. As a result, inflammatory responses were inhibited.

In summary, this integrated metabolomics approach provided ample evidences demonstrating substantial amelioration in cerebral I/R injury by Ber, Bai, and Jas.  Figure 11 depicts how HLJDD and its three principal components affected the metabolic profiles and exhibited the therapeutic effects on ischemic stroke, which may be attributable to the amelioration of disordered metabolism, the upregulation of neuron autophagy, and the downregulation of oxidative stress and inflammatory response. Amelioration of neurological function in I/R by Ber and Bai may due to their induced increases in NF-*κ*B, iNOS, and COX-2 protein expression. In addition, the neuroprotective effects of Ber and Jas were related to their regulation of oxidative stress and autophagy. The results of the present study indicate that Ber, Bai, and Jas of HLJDD are responsible for the effectiveness of HLJDD on the treatment of ischemic stroke and thus are of great potential for the drug development of ischemic brain injury based on them.

## Supplementary Material

Supplementary Table S1. Primers used for real-time PCR assays performed on the LC480 system. Supplementary Table S2. Compounds detected in the extracts of HLJDD obtained by HPLC-Q-TOF-MS. Supplementary Table S3. Compounds detected in the extracts of HLJDD obtained by HPLC-Q-TOF-MS. Supplementary Table S4. Compounds detected in the extracts of HLJDD obtained by HPLC-Q-TOF-MS. Supplementary Table S5. Compounds detected in the extracts of HLJDD obtained by HPLC-Q-TOF-MS. Supplementary Table S6. Compounds detected in the extracts of HLJDD obtained by HPLC-Q-TOF-MS. Supplementary Table S7. The assignment of metabolites in serum of all groups. Supplementary Table S8. The assignment of metabolites in brain extracts of all groups. Supplementary Table S9. Calibration curve, correlation coefficient (r^2^), test range and instrumental LOD, LOQ for berberine, baicalin and jasminoidin in methanol solution (n=6). Supplementary Table S10. Precision, accuracy and recovery of berberine, baicalin and jasminoidin (n=6). Supplementary Table S11. Calibration curve, correlation coefficient (r^2^), test range, weight cofficient and LLOQ for berberine, baicalin and jasminoidin in methanol solutions (n=6). Supplementary Table S12. Precision and accuracy of berberine, baicalin and jasminoidin for QC samples in rat plasma (n=6). Supplementary Table S13. Recovery and matrix effect for berberine, baicalin and jasminoidin in rat plasma (n=6). Supplementary Table S14. Stability of berberine, baicalin and jasminoidin in plasma under various storage conditions (n=6). Supplementary Table S15. Stability of berberine, baicalin and jasminoidin in brain extracts under various storage conditions (n=6). Supplementary Figure S1. Score plots according to OPLS-DA analysis based on ^1^H NMR data from serum of all groups at each time point. Supplementary Figure S2. Score plots according to OPLS-DA analysis based on ^1^H NMR data from brain extracts of all groups at each time point. Supplementary Figure S3. Profile of Profile of UV chromatogram and TIC and chromatogram. Supplementary Figure S4. Quantitative analysis of regional cerebral blood flow (rCBF) in different groups. Supplementary Figure S5. Typical 500 MHz ^1^H NMR spectra of serum and brain tissues. Supplementary Figure S6. Fold change plots color-coded with p-values adjusted by Benjamini-Hochberg method indicating significance of altered metabolites in serum and brain extracts. Supplementary Figure S7. Brain and plasma concentration versus time profiles of berberine, baicalin and jasminoidin after administration in rats.
