# Supplementary material for: Treatment Effects of Ischemic Stroke by Berberine, Baicalin, and Jasminoidin from Huang-Lian-Jie-Du-Decoction (HLJDD) Explored by an Integrated Metabolomics Approach
Source: Oxid Med Cell Longev. 2017 Aug 14;2017:9848594. doi: 10.1155/2017/9848594 (PMC5574319; doi:10.1155/2017/9848594)
Supplement: Supplementary file 1 — Supplementary Table S1. Primers used for real-time PCR assays performed on the LC480 system. Supplementary Table S2. Compounds detected in the extracts of HLJDD obtained by HPLC-Q-TOF-MS. Supplementary Table S3. Compounds detected in the extracts of HLJDD obtained by HPLC-Q-TOF-MS. Supplementary Table S4. Compounds detected in the extracts of HLJDD obtained by HPLC-Q-TOF-MS. Supplementary Table S5. Compounds detected in the extracts of HLJDD obtained by HPLC-Q-TOF-MS. Supplementary Table S6. Compounds detected in the extracts of HLJDD obtained by HPLC-Q-TOF-MS. Supplementary Table S7. The assignment of metabolites in serum of all groups. Supplementary Table S8. The assignment of metabolites in brain extracts of all groups. Supplementary Table S9. Calibration curve, correlation coefficient (r2), test range and instrumental LOD, LOQ for berberine, baicalin and jasminoidin in methanol solution (n=6). Supplementary Table S10. Precision, accuracy and recovery of berberine, baicalin and jasminoidin (n=6). Supplementary Table S11. Calibration curve, correlation coefficient (r2), test range, weight cofficient and LLOQ for berberine, baicalin and jasminoidin in methanol solutions (n=6). Supplementary Table S12. Precision and accuracy of berberine, baicalin and jasminoidin for QC samples in rat plasma (n=6). Supplementary Table S13. Recovery and matrix effect for berberine, baicalin and jasminoidin in rat plasma (n=6). Supplementary Table S14. Stability of berberine, baicalin and jasminoidin in plasma under various storage conditions (n=6). Supplementary Table S15. Stability of berberine, baicalin and jasminoidin in brain extracts under various storage conditions (n=6). Supplementary Figure S1. Score plots according to OPLS-DA analysis based on 1H NMR data from serum of all groups at each time point. Supplementary Figure S2. Score plots according to OPLS-DA analysis based on 1H NMR data from brain extracts of all groups at each time point. Supplementary Figure S3. Profile of [file 9848594.f1.pdf]

# Treatment effects of ischemic stroke by berberine, baicalin and jasminoidin from Huang-Lian-Jie-Du-Decoction (HLJDD) explored by integrated metabolomics approach

Qian Zhang[1], Xiaowei Fu[1], Junsong Wang[2 *], Minghua Yang[1], and Lingyi Kong[1 *]

[1]State Key Laboratory of Natural Medicines, Department of Natural Medicinal Chemistry, China Pharmaceutical University, 24 Tong Jia Xiang, Nanjing, 210009, P.R. China

[2]Center for Molecular Metabolism, Nanjing University of Science and Technology, 200 Xiao Ling Wei Street, Nanjing, 210094, P.R. China

[*] Correspondence and requests for materials should be addressed to Lingyi Kong (email: cpu_lykong@126.com) or Junsong Wang (email: wang.junsong@gmail.com)

# Supplementary Methods

## Herbal Materials and Samples Preparations

HLJDD, composed of *Rhizoma Coptidis* (*Coptis chinensis* Franch, Ranunculacea), *Radix Scutellariae* (*Scutellaria baicalensis* Georgi, Labiatae), *Cortex Phellodendri* (*Phellodendron chinensis* Schneid, Rutaceae) and *Fructus Gardeniae* (*Gardenia jasminoides* Ellis, Rubiaceae), in a ratio of 3:2:2:3 (w/w/w/w), reaching a total weight of 1.0 kg, was extraction with 70 % ethanol (1:10, w/v) under reflux three times for 1h each. The extract solution was combined and freeze-dried in a vacuum to collect HLJDD extract (264.8 g, yield: 26.48 %). All herbs were purchased from Jiangsu Medicine Company (Nanjing, China, Drug GMP certificate: SUJ0623; Drug Manufacturing Certificate: SUY20110051), and authenticated by Professor Mian Zhang, Department of Medicinal Plants, China Pharmaceutical University, Nanjing, China.

Berberine (purity > 98.0%) and jasminoidin (purity > 98.0%) were purchased from Qingdao Jieshikang Biotech Co., Ltd. (Qingdao, China), and baicalin (purity > 98.0%) was purchased from Dalian Meilun Biotech Co., Ltd. (Dalian, China). Sodium 3-trimethylsilyl-1-(2, 2, 3, 3-2H4) propionate (TSP) was provided by Sigma Chemical Co. (St. Louis, MO, USA). Deuterium oxide (D2O, 99.9%) was purchased from Aldrich (Steinheim, Germany). Ultra-pure distilled water was prepared using a Milli-Q purification system. Acetonitrile (HPLC grade) was obtained from Merck KGaA (Darmstadt, Germany). Acetonitrile and formic acid (LC-MS grade) were obtained from ROE Scientific Inc. (Newark USA).

**Quantitative analysis**

Linearity (Supplementary Table S9) was evaluated by analyzing the standard solutions of Ber, Bai and Jas at seven different concentration levels. Based on the obtained results, the linearities of the analytical response across the studied range were excellent, with correlation coefficients ($r^2$) at 0.9994, 0.9992 and 0.9991, respecively. Sensitivity of the method was evaluated by determining limits of detection (LOD) and the pre-determined values of quantitation limits (LOQ). LOD and LOQ were defined at the concentration with a signal-to-noise ratio (determined by peak height) of at least 3 and 10, respectively. The parameter was determined empirically by triplicate analysis of a series of concentrations of standard solution.

Method precision was checked by intra-day and inter-day variability. The intra-day variability study was conducted by the injection of the same standard solution at six consecutive times in the same day. The inter-day variability study was conducted for three successive days using the same solution. The precision were expressed in terms of relative standard deviation (RSD). The RSD values obtained from run-to-run and day-to-day precision studies were summarized in Supplementary Table S10. Based on our results, the developed method was precise.

The recovery rate was performed by adding a known amount of standards into a certain amount of HLJDD extract. The mixture was extracted and analysed using the method mentioned above. Three replicates were performed for the test. The recovery of Ber, Bai and Jas was summarized in Supplementary Table S10.

**Sample collection**

The rats were deeply anesthetized with 3.5% chloral hydrate (350 mg/kg body weight) 24 hours after reperfusion and then sacrificed. Blood was collected from the abdominal aorta, and the serum samples were obtained by centrifugation at 2,000 ×*g* for 10 min and were stored at −80 °C before the experiments. Brain tissues were quickly removed, weighed, and rinsed with cold phosphate-buffered saline (PBS). An aliquot of brain samples was frozen stored at −80 °C for $^{1}$H NMR studies, and another brain portion used for histological examination was fixed in neutral buttered formalin (10% formalin in 0.08 M sodium phosphate, pH 7.4).

**$^{1}$H NMR spectroscopic measurement of serum and brain tissues**

After thawing, serum samples (300 μl) were added to 300 μl $D_2O$ (0.2 mol $L^{-1}$ $Na_2HPO_4$ and 0.2 mol $L^{-1}$ $NaH_2PO_4$, pH 7.4, containing 0.05 % TSP). TSP acted as a chemical shift reference (δ 0.0) and $D_2O$ provided a lock signal. The samples were vortexed and centrifuged at 12,000 ×*g* for 10 min at 4 ℃ to remove insoluble material. The supernatants (550 μL) were then pipetted into 5-mm NMR tubes for NMR recording. Frozen brain tissues (200-300 mg) were homogenized in a mixture of volumetric equivalent acetonitrile and water (5 mL/g tissue) in an ice/water bath and centrifuged at 12,000 ×*g* for 10 min at 4 ℃. The supernatant was collected and concentrated under a stream of nitrogen and lyophilized. Dried brain extracts were reconstituted in 600 μL $D_2O$ (0.2 M $Na_2HPO_4$ and 0.2 M $NaH_2PO_4$, pH 7.0, containing 0.05 % TSP). The supernatants were then pipetted into 5-mm NMR tubes for NMR recording.

$^1$H NMR spectra of the samples were recorded on a Bruker AV 500 MHz spectrometer at 300 K. For each serum sample, the transverse relaxation-edited Carr-Purcell-Meiboom-Gill (CPMG) spin-echo pulse sequence (RD-90°-(τ-180°-τ) n-ACQ) with a total spin-echo delay (2nt) of 40 ms was used to suppress broad signals from macromolecules; therefore, the micromolecules signals were clearly observed. $^1$H NMR spectra were measured with 128 scans into 32K data points over a spectral width of 10,000 Hz. Prior to Fourier transformation, an exponential window function with a line broadening of 0.3 Hz was used for the free induction decays (FIDs). For the brain, a nuclear overhauser effect spectroscopy (NOESYPR) pulse sequence (relaxation delay-90°-μs-90°-tm-90°-acquire-FID) was used to attenuate the residual water signal. FIDs were collected into 32K data points over a spectral width of 10,000 Hz with an acquisition time of 2.04 s. The FIDs were weighted by an exponential function with a 0.3 Hz line-broadening factor prior to Fourier transformation.

**Spectral pre-processing**

The spectra for all samples were manually phased and baseline corrected and referenced to TSP at 0.0 ppm, using Bruker Topspin 3.0 software (Bruker GmbH, Karlsruhe, Germany). The $^1$H NMR spectra were automatically exported to ASCII files using MestReNova (Version 8.0.1, Mestrelab Research SL), which were then imported into "R" (http://cran.r-project.org/) and aligned with an in-house developed R-script to further reduce phase and baseline distortions. The one-dimensional (1D) spectra were converted to an appropriate format for statistical analysis by

automatically segmenting each spectrum into 0.015-ppm integrated spectral regions (buckets) between 0.2 and 10 ppm. The region of the residual water and affected signals (4.70-9.70 for serum and 4.65-5.25 for brain extracts) was removed. To account for different dilutions of samples, all binned spectra were probability quotient normalized and then mean-centred before further multivariate analysis.

**Data analysis**

The mean-centred and Pareto-scaled NMR data were analysed by principal component analysis (PCA) and OPLS-DA. PCA is an exploratory unsupervised method to maximize the separation by providing model-free approaches for determining the latent or intrinsic information in the dataset. However, no clustering was observed when variables were not selected. OPLS-DA determined PLS components that are orthogonal to the grouping and was used to concentrate group discrimination into the first component with remaining unrelated variations contained in subsequent components. All OPLS-DA models were validated by a repeated two-fold cross-validation method and permutation test (2000 times)[1]. The parameters $R^2$ and $Q^2$ reflected the goodness of fitness and the predictive ability of the models, respectively. The p-value of the permutation test denoted the number of times that the permutated data yielded a better result than the one using the original labels. The fold change values of metabolites among different groups were calculated. The Benjamini & Hochberg method[2] was used to adjust the p-values for controlling the false discovery rate in multiple comparisons using scripts written in R language.

**Real-time quantitative RT-PCR analysis**

Total RNA was extracted from ischemic hippocampus tissue using RNAiso Plus reagent (TaKaRa Biotechnology Co., Ltd, Dalian, China) following the manufacture's protocol. The quantitative real-time polymerase chain reaction (qRT-PCR) was performed with a LightCycler 480 (Roche Molecular Biochemicals, Mannheim, Germany). RNA was quantified by measuring absorption at 260 nm, and 1 μg RNA was reverse transcribed to cDNA by using the Transcriptor First Strand cDNA Synthesis Kit (Roche Diagnostics, Basel, Switzerland) [3]. Thermal cycling conditions included an initial denaturation at 95 ℃ for 5 min, followed by 40 cycles of denaturation (10 s at 95 ℃), annealing (15 s at 60 ℃) and extension (15 s at 72 ℃ with a single fluorescence measurement), a melting curve programme (60-95 ℃ with a 0.11 ℃ /s heat increase and continuous fluorescence measurement) and a cooling step to 40 ℃. The $2^{-\Delta\Delta CT}$ method was used for the calculation of the relative differences in mRNA abundance. The relative gene expression level of each gene was normalized to $\beta$-actin levels. The results are expressed as fold changes. Forward and reverse primers used in the present study are listed in Supplementary Table S1.

**Western blot analysis**

Western blot analysis was performed at 24 h after reperfusion. Cell proteins in ischemic penumbra were extracted from the fresh cerebral cortex. Briefly, the samples were homogenized in 1xRIPA lysis buffer (50 mM Tris-HCl, pH 7.4, 150 mM NaCl, 0.25% deoxycholic acid, 1% NP-40, 1 mM EDTA, and phosphatase and protease inhibitors) (Amresco, Solon, USA) to extract the total proteins. Cytosolic and nuclear

extractions were performed using a cytosolic/nuclei isolation kit (Beyotime Biotechnology Co., Ltd., Nanjing, China), according to the manufacturer's protocols. Protein concentrations were measured using the bicinchoninic acid protein (BCA) assay kit (Beyotime, Haimen, China). Equal volumes and quantities of protein solutions were separated by polyacrylamide gel electrophoresis (PAGE, BioRad Laboratories, Hercules, CA) and electrotransferred onto the poly-vinylidnene fluoride membranes (BioRad Laboratories, Hercules, CA). The membranes were blocked in 5% non-fat dry milk in Tris-buffered saline containing 0.05% Tween-20 (TBST), incubated in primary antibody at 4 ℃ overnight, rinsed three times with TBST for 10 min, and finally incubated with horseradish peroxidase-conjugated secondary anti-rabbit antibody for 2 h. The following primary antibodies were used: pp65, LC3, p62, PARP, Atg-3, Atg-5, Atg-7, Atg-12, Beclin-1, phospho-mTOR, phospho-PI3k, phospho-Akt, phospho GSK-3β, β-actin and PCNA, probed with secondary isotype specific antibodies tagged with horseradish peroxidase (Cell Signaling Technology). Chemiluminescence substrate (ECL Plus) was used to incubate the blots and band intensities were analysed using the ChemiDOC™ XRS+ system (Bio-Rad Laboratories, Hercules, CA, USA). PCNA and β-actin proteins were considered as loading controls.

**Pharmacokinetic study**

Blood samples, ca. 300 μL, were collected at 15 min after drug administration and at 1 day, 3 day and 7 day after reperfusion. Plasma was harvested by centrifuging the blood sample at 4 ℃ and 2,000 × g for 10 min, and then stored at −80 ℃ until

analysis. After anesthetization, the animals were then sacrificed at 1day, 3day and 7day after reperfusion. Brains were obtained at the time of death and quickly rinsed with normal saline, and then stored at −80 ℃ for analysis. Brains were accurately weighed (200 mg) and homogenized in 600 μL of deionized water under ice bath using a Potter-Elvehjem homogenizer.

Plasma sample/brain homogenate (100 μL) was spiked with 20 μL of 1-hydroxytacrine (IS) working solution (5 ng/mL) and mixed for 10 s, to which, 400 μL of methanol was added, and the mixture was vortexed for 3 min. The well-vortexed solution was then centrifuged at 14,000 × g for 5 min and 400 μL of the supernatant was transferred into a new centrifuge tube and dried. The residue was reconstituted in 100 μL methanol, centrifuged at 14,000 × g for 5 min, and 5 μL of the supernatant was injected into the HPLC-QTOF-MS/MS system for analysis.

Chromatographic separation was performed on an Agilent ZORBAX SB-C18 column (3.5 mm × 10 mm, 5 μm, Agilent Corporation, Santa Clara, CA, USA) using a mobile phase of acetonitrile (B) and $H_2O$ (A, with 0.1% formic acid) (injection volume, 5 μl; flow rate, 1 mL/min; temperature, 35 ℃). The mobile phase gradient program was used as follows: 0-2 min, 10-25% B; 2-3 min, 25-30% B; 3-5 min, 30-70% B; 5-6 min, 70-90% B; 6-8 min, 90% B, 8-10 min, 90-10% B. Online HPLC-QTOF-MS/MS analysis was made using an Agilent 1290 infinity LC system equipped with a binary pump, degasser, column oven, autosampler and diode array detector, which was coupled with a 6520 quadruple time-of-flight tandem mass spectrometer. The positive ion mode were conducted using conditions as follows:

skimmer, 75 V; drying gas ($N_2$) flow rate, 10 L/min; drying gas temperature, 320 ℃; nebulizer, 45 psi; capillary voltage, 4000 V; fragmentor, 180 V; collision energy, 30eV (for berberine and jasminoidin) and 20ev (for baicalin and IS). All the operation, acquisition, and analysis of data were made by Agilent LC-Q-TOF-MS Mass Hunter Acquisition Software Version B.04.00 (Agilent Technologies). The detection of the ions was performed in the targeted MSMS mode, monitoring the *m/z* 336.1230 precursor ion $[M+H]^+$ to the *m/z* 320.0931 product ion for berberine; the *m/z* 447.0922 precursor ion $[M+H]^+$ to the *m/z* 271.0595 product ion for baicalin; the *m/z* 411.1262 precursor ion $[M+H]^+$ to the *m/z* 217.0803 product ion for jasminoidin, and the *m/z* 215.1238 precursor ion $[M+H]^+$ to the *m/z* 197.0599 product ion for IS.

Stock solutions of berberine, baicalin, jasminoidin and IS were prepared by dissolving accurate amounts of reference standards in methanol at a concentration of 1.0 mg/mL, stored at 4 ℃. Series of working solutions of berberine, baicalin, jasminoidin were prepared by serial dilution of the stock solution with methanol. The working solution of IS (5 ng/mL) was obtained by diluting the stock solution in methanol.

Calibration standards for berberine, baicalin, jasminoidin were prepared by spiking 100 μL of blank plasma or brain homogenate with 10 μL of corresponding working solutions to yield final concentrations of 5, 10, 20, 50, 100, 200, 500, 1000 and 2000 ng/mL in rat plasma and 2, 5, 10, 20, 50, 100, 200, 500, 1000 and 2000 ng/mL in brain homogenate. QC samples were prepared in the same manner at concentrations of 4, 800 and 1600 ng/mL.

The selectivity was investigated by analyzing blank plasma and brain samples from six rats for interference. Calibration curve ($y = ax + b$) was acquired by plotting the peak area ratio of berberine, baicalin, jasminoidin to IS ($y$) against the corresponding nominal berberine, baicalin, jasminoidin concentration ($x$) by weighted ($1/x^2$) least-squares linear regression. The lower Limit of quantitation (LLOQ) was defined as the lowest concentration where the signal-to-noise (S/N) ratio was larger than 10 and both the precision (the relative standard deviation, RSD) and accuracy (relative error, RE) were less than or equal to 20% by analyzing the six replicates of samples spiked with each analyte (Supplementary Table S11). With the established chromatographic conditions, berberine, baicalin, jasminoidin and IS were baseline and well separated from each other with no interference from endogenous materials in rat plasma and brain tissue. The retention time for berberine, baicalin, jasminoidin and IS were 4.795, 4.468, 2.857 and 5.603 min, respectively. Linear regressions of the peak area ratios versus concentrations were fitted over the concentration range of 5-2000 ng/mL and 2-2000 ng/mL for berberine, baicalin, jasminoidin in rat plasma and brain extracts, respectively.

The intra-day accuracy and precision were determined within 1 day by analysis of six replicates of QC samples at 4, 800 and 1600 ng/mL. The inter-day precision and accuracy were determined in 3 consecutive days. The accuracy and precision were expressed in terms of RE and RSD, respectively. The precision should not exceed 15% and accuracy should be within ± 15% for the QC samples. The intra-day and inter-day and precision of the assay method for all analytes in rat plasma and brain

tissue were shown in Supplementary Table S12.

The recovery was assessed by comparing the mean peak area of QC samples extracted from biological matrix with the peak area of reference standards prepared in reconstitute solvent. The matrix effect was evaluated by comparing the peak areas of analytes in the above mentioned standard solutions to those of the neat standards at the same concentration. The stability of berberine, baicalin and jasminoidin in rat plasma and brain extracts was investigated by analysis of three levels of QC samples stored at −80 ºC for two weeks (long-term stability), at 25 ℃ for 4 h (short-term stability) and after three freeze-thaw cycles (−80 ºC to 25 ºC). The data for the absolute recovery and matrix effect were summarized in Supplementary Table S13. All matrix effects were considered acceptable in this method. The data for short-term stability, long-term stability, freeze-thaw stability and post-preparative stability were shown in Supplementary Tables S14 and 15, also acceptable for routine analysis.

Brain/plasma disposition of berberine, baicalin and jasminoidin

The developed and validated method was applied to determine berberine, baicalin and jasminoidin in rat plasma and brain tissues after administration. The mean concentration-time curves of berberine, baicalin and jasminoidin in plasma and brain were shown in Supplementary Figure S7.

## Supplementary Table

**Supplementary Table S1** Primers used for real-time PCR assays performed on the LC480 system

| Genes | Forward primer (5'-3') | Reverse primer (5'-3') |
|---|---|---|
| TNF-α | CATCCGTTCTCTACCCAGCC | AATTCTGAGCCCGGAGTTGG |
| IL-1β | CACCGAAGTGACCATTGGCT | AAGCTGTAATGGTCTGCCTCC |
| IL-2 | CGTGATGTACCTCCGTGCTT | ATTCACGGTGCAGCTTCTCA |
| IL-6 | CACTTCACAAGTCGGAGGCT | TCTGACAGTGCATCATCGCT |
| iNOS | GCCACAGTCCTCTTTGCTACT | GGGGCAGTCTCCATTCGC |
| COX-2 | CTGCGACTCCTTGACGTTGA | AAGCCTAATGTGGGGACAGC |

TNF-α: Tumor Necrosis Factor-α; IL-1β: interleukin-1β; IL-2: interleukin-2; IL-6: interleukin-6

**Supplementary Table S2** Compounds detected in the extracts of HLJDD obtained by HPLC-Q-TOF-MS

| Peak | $t_R$ (min) | Experimental $M^+$ | Calculated $M^+$ | Error (ppm) | Molecular formula | Proposed compound |
|---|---|---|---|---|---|---|
| **1** | 10.741 | 342.1704 | 342.1700 | -0.13 | $C_{20}H_{24}NO_4$ | Phellodendrine |
| **2** | 11.117 | 342.1701 | 342.1700 | 0.38 | $C_{20}H_{24}NO_4$ | Magnoflorine |
| **3** | 11.767 | 342.1699 | 342.1700 | 0.66 | $C_{20}H_{24}NO_4$ | Cyclanoline |
| **4** | 15.652 | 322.1070 | 322.1074 | 1.02 | $C_{19}H_{16}NO_4$ | Berberrubine |
| **5** | 17.158 | 338.1392 | 338.1387 | -0.25 | $C_{20}H_{19}NO_4$ | Jatrorrhizine / Columbamin |
| **6** | 17.398 | 338.1384 | 338.1387 | 0.94 | $C_{20}H_{19}NO_4$ | Jatrorrhizine / Columbamin |
| **7** | 17.550 | 336.1232 | 336.1230 | 0.26 | $C_{20}H_{17}NO_4$ | Epiberberine |
| **8** | 17.714 | 338.1386 | 338.1387 | 0.67 | $C_{20}H_{19}NO_4$ | Jatrorrhizine isomers |
| **9** | 17.812 | 320.0918 | 320.0917 | 0.32 | $C_{19}H_{14}NO_4$ | Coptisine |
| **10** | 18.664 | 350.1388 | 350.1387 | 0.4 | $C_{21}H_{20}NO_4$ | Jatrorrhizine |
| **11** | 19.335 | 352.1540 | 352.1543 | 0.92 | $C_{21}H_{22}NO_4$ | Palmatine isomers |
| **12** | 19.618 | 352.1545 | 352.1543 | 0.32 | $C_{21}H_{22}NO_4$ | Palmatine |
| **13** | 20.050 | 336.1235 | 336.1230 | -0.18 | $C_{20}H_{18}NO_4$ | Berberine |

**Supplementary Table S3** Compounds detected in the extracts of HLJDD obtained by HPLC-Q-TOF-MS

| Peak | $t_R$ (min) | Experimental $[M+H]^+$ | Calculated $[M+H]^+$ | Error (ppm) | Molecular formula | Proposed compound |
|---|---|---|---|---|---|---|
| **14** | 12.416 | 369.1186 | 369.1180 | 1.14 | $C_{17}H_{20}O_9$ | Chlorogenic acid methylester |
| **15** | 12.727 | 314.1750 | 314.1751 | 0.64 | $C_{19}H_{23}NO_3$ | Armepavine |
| **16** | 15.246 | 368.1183 | 368.1180 | 0.75 | $C_{17}H_{20}O_9$ | Methylchlorogenate |
| **17** | 16.094 | 352.1183 | 352.1179 | -0.02 | $C_{20}H_{17}NO_5$ | Berlambine |
| **18** | 16.579 | 549.1609 | 549.1603 | -0.07 | $C_{26}H_{28}O_{13}$ | Chrysin 6-C-glucoside 8-C-arabinoside |
| **19** | 16.765 | 549.1607 | 549.1603 | 0.95 | $C_{26}H_{28}O_{13}$ | Chrysin 6-C-arabinoside 8-C-glucoside |
| **20** | 16.770 | 336.1227 | 336.1230 | 0.96 | $C_{20}H_{17}NO_4$ | Berberine isomers |
| **21** | 24.955 | 447.0925 | 447.0922 | 0.55 | $C_{21}H_{18}0_{11}$ | Baicalin |
| **22** | 29.555 | 447.0923 | 447.0922 | 0.35 | $C_{21}H_{18}O_{11}$ | Baicalin isomers |
| **23** | 31.186 | 461.1080 | 461.1078 | 0.35 | $C_{22}H_{20}O_{11}$ | Oroxylin A 7-O-glucuronide |
| **24** | 31.481 | 431.0971 | 431.0973 | 0.7 | $C_{21}H_{18}O_{10}$ | Chrysin-7-O-glucoronide |
| **25** | 31.928 | 477.1028 | 477.1032 | 0.65 | $C_{22}H_{20}O_{12}$ | 5,6,7-trihydroxy-8-methoxyflavon-7-O-glucoronide |

| | | | | | | |
|---|---|---|---|---|---|---|
| **26** | 32.436 | 461.1082 | 461.1078 | 0.15 | $C_{22}H_{20}O_{11}$ | Wogonoside |
| **27** | 36.179 | 471.2012 | 471.2013 | 0.42 | $C_{26}H_{30}O_{8}$ | Obaculactone |
| **28** | 36.992 | 285.0755 | 285.0757 | 0.75 | $C_{16}H_{12}O_{5}$ | Wogonin |
| **29** | 37.014 | 375.1082 | 375.1074 | 0.78 | $C_{19}H_{18}O_{8}$ | 5,7-Dihydroxy-6,8-dimet hoxyflavone |
| **30** | 37.330 | 352.1182 | 352.1179 | 0.11 | $C_{20}H_{17}NO_{5}$ | 8-Oxyberberine |
| **31** | 37.543 | 285.0755 | 285.0757 | 0.9 | $C_{16}H_{12}$O5 | Wogonin isomers |
| **32** | 43.300 | 285.0766 | 285.0757 | -0.91 | $C_{16}H_{12}O_{5}$ | Oroxylin A |

**Supplementary Table S4** Compounds detected in the extracts of HLJDD obtained by HPLC-Q-TOF-MS

| Peak | $t_R$ (min) | Experimental $[M+Na]^+$ | Calculated $[M+Na]^+$ | Error (ppm) | Molecular formula | Proposed compound |
|---|---|---|---|---|---|---|
| **33** | 13.213 | 411.1259 | 411.1262 | 0.83 | $C_{17}H_{24}O_{10}$ | Jasminoidin |

**Supplementary Table S5** Compounds detected in the extracts of HLJDD obtained by HPLC-Q-TOF-MS

| Peak | $t_R$ (min) | Experimental $[M-H]^-$ | Calculated $[M-H]^-$ | Error (ppm) | Molecular formula | Proposed compound |
|---|---|---|---|---|---|---|
| **34** | 11.710 | 353.0875 | 353.0878 | 0.15 | $C_{16}H_{18}O_{9}$ | Chlorogenic acid |
| **14** | 12.474 | 367.1028 | 367.1035 | 1.45 | $C_{17}H_{20}O_{9}$ | Chlorogenic acid methylester |
| **35** | 15.295 | 367.1031 | 367.1035 | 0.93 | $C_{17}H_{20}O_{9}$ | 3-O-Feruloylquinic acid |
| **36** | 16.730 | 547.1455 | 549.1457 | 0.7 | $C_{26}H_{28}O_{13}$ | Puerarin 6"-O-xyloside |
| **20** | 17.034 | 547.1455 | 547.1457 | 0.7 | $C_{26}H_{28}O_{13}$ | Chrysin 6-C-arabinoside 8-C-glucoside |
| **37** | 18.263 | 461.0719 | 461.0725 | 1.22 | $C_{21}H_{18}O_{12}$ | 5,7-dihydroxy-2’-methoxy flavon-7-O- glucoronide |
| **38** | 19.802 | 695.2186 | 695.2193 | 1.01 | $C_{32}H_{40}O_{17}$ | 6”-*0*-trans-p-coumaroylge-nipin gentiobioside |
| **39** | 21.726 | 551.2133 | 551.2134 | 0.65 | $C_{27}H_{36}O_{12}$ | Quercetin-3-O-rutinose |
| **21** | 25.073 | 445.0774 | 445.0776 | 0.05 | $C_{21}H_{18}O^{11}$ | Baicalin |
| **23** | 29.836 | 445.0771 | 445.0776 | 1.06 | $C_{21}H_{18}O_{11}$ | Baicalin isomers |
| **24** | 31.703 | 429.0824 | 429.0827 | 0.91 | $C_{21}H_{18}O_{10}$ | Chrysin-7-O-glucoronide |
| **40** | 32.074 | 475.0877 | 475.0882 | 1.05 | $C_{22}H_{20}O_{12}$ | 5,6,7-trihydroxy-8-methox yflavon-7-O- glucoronide |
| **41** | 32.505 | 459.0930 | 459.0933 | 0.82 | $C_{22}H_{20}O_{11}$ | Wogonoside isomers |

| | | | | | | |
|---|---|---|---|---|---|---|
| **42** | 33.045 | 697.2707 | 697.2713 | 0.96 | $C_{33}H_{46}O_{16}$ | Luteolin-7-O-glucuronide |
| **43** | 33.334 | 459.0933 | 459.0933 | 0.44 | $C_{22}H_{20}O_{11}$ | Oroxylin A 7-O-glucuronide isomers |
| **44** | 33.591 | 473.1081 | 473.1089 | 1.34 | $C_{23}H_{22}O_{11}$ | Baicalein-7-O-glucuronide ethyl ester |
| **45** | 34.638 | 491.1187 | 491.1195 | 1.05 | $C_{23}H_{24}O_{12}$ | 5,2,6'-trihydroxy-7,8-dimethoxyflavon-2'-O-β-D-glucopyranoside |
| **46** | 37.012 | 283.0609 | 283. 0612 | 1.22 | $C_{16}H_{12}O_5$ | Oroxylin A isomers |
| **29** | 37.105 | 373.0927 | 373.0929 | 0.4 | $C_{19}H_{18}O_8$ | 5,7-dihydroxy-6,8-dimethoxyflavone |
| **31** | 37.634 | 283.0606 | 283.0612 | 1.97 | $C_{16}H_{12}O_5$ | Wogonin isomers |

**Supplementary Table S6** Compounds detected in the extracts of HLJDD obtained by HPLC-Q-TOF-MS

| **Peak** | **$t_R$ (min)** | **Experimental [M+COOH]$^-$** | **Calculated [M+COOH]$^-$** | **Error (ppm)** | **Molecular formula** | **Proposed compound** |
|---|---|---|---|---|---|---|
| **47** | 11.121 | 595.1873 | 595.1880 | 1.02 | $C_{23}H_{34}O_{15}$ | Genipin-1-β-D-gentiobioside |
| **33** | 13.058 | 433.1350 | 433.1350 | 0.67 | $C_{17}H_{24}O_{10}$ | Jasminoidin |

**Supplementary Table S7** The assignment of metabolites in serum of all groups

| No. | Metabolites | Assignments | Chemical shift(ppm) |
|---|---|---|---|
| 1 | Isoleucine | δ$CH_3$, γ$CH_3$, α$CH$ | 0.93(t), 1.0(d), 1.46(m) |
| 2 | Leucine | δ$CH_3$, δ$CH_3$, γCH, αCH | 0.94(t), 0.96(t), 1.71(m), 3.74(m) |
| 3 | Valine | γ$CH_3$, γ$CH_3$ | 0.98(d), 1.03(d), 2.27(m), 3.60(d) |
| 4 | 3-Hydroxybutyrate | γ$CH_3$, βCH, α$CH_2$ | 1.25(d), 2.31(m), 2.41(m), 4.16(m) |
| 5 | Lactate | $CH_3$, CH | 1.37(d), 4.11(q) |
| 6 | Alanine | β$CH_3$, αCH | 1.53(d), 3.78(q) |
| 7 | Lysine | δ$CH_2$ | 1.48(m), 1.73(m), 1.91(m), 3.03(t), 3.76(t) |
| 8 | Arginine | γ$CH_2$, β$CH_2$ | 1.78(m), 1.95(m) |
| 9 | Acetoacetate | $CH_3$, $CH_2$ | 2.28(s), 3.44(s) |
| 10 | Glutamate | β$CH_2$, β$CH_2$, γ$CH_2$, αCH | 2.10(m), 2.14(m), 2.36(m), 2.50(m), 3.77(t) |
| 11 | Pyruvate | β$CH_3$ | 2.41(s) |
| 12 | Succinate | $CH_2$ | 2.41(s) |
| 13 | Glutamine | β$CH_2$, γ$CH_2$, αCH | 2.46(m), 3.77(t) |
| 14 | Citrate | 1/2$CH_2$, 1/2$CH_2$ | 2.59(d), 2.73(d) |
| 15 | NADPH | $CH_2$ | 2.80 (dt) |
| 16 | Cysteine | CH2, CH | 3.06(m), 3.97(dd) |
| 17 | Creatine | $CH_2$, $CH_3$ | 3.08(s), 3.93(s) |
| 18 | TMAO | $CH_3$ | 3.34(s) |
| 19 | Taurine | $NH_2$-$CH_2$, $SO_3$-$CH_2$ | 3.25(t), 3.43(t) |
| 20 | Glycine | CH2 | 3.57(s) |
| 21 | Glycerol | $CH_2$, $CH_2$, CH | 3.55(ABX), 3.68(ABX), 3.79(ABX) |
| 22 | Acetylcholine | $N(CH_3)_3$, N-$CH_2$, O-$CH_2$, $CH_3$ | 3.21(s), 3.67(t), 4.51(t), 2.1(s) |
| 23 | Betaine | $N(CH_3)_3$, $CH_2$ | 3.28(s), 3.90(s) |
| 24 | α-Glucose | 2-CH, 4-CH, 5-CH, 3-CH, 6-CH, 6-CH, 1-CH | 3.65-3.92 (m) |
| 25 | β-Glucose | 1-CH, 2-CH, 3-CH, 4-CH, 5-CHC | 5.24(dd), 3.54(dd), 3.73(dd), 3.42(t), 3.84(m) |
| 26 | OPC | N(CH3)3, N-CH2, O-CH2 | 3.21(s), 3.57(t), 4.16(t) |

Multiplicity: singlet (s), doublet (d), triplet (t), doublet of doublets (dd), quartets (q), multiplet (m).

**Supplementary Table S8** The assignment of metabolites in brain extracts of all groups.

| No. | Metabolite | Assignments | Chemical shift |
|---|---|---|---|
| 1 | Isoleucine | $\delta CH_3$, $\gamma CH_3$, $\alpha CH$ | 0.93(t), 1.0(d), 1.46(m) |
| 2 | Leucine | $\delta CH_3$, $\delta CH_3$, $\gamma CH$, $\alpha CH$ | 0.94(t), 0.96(t), 1.71(m), 3.74(m) |
| 3 | Valine | $\gamma CH_3$, $\gamma CH_3$ | 0.98(d), 1.03(d), 2.26(m), 3.60(d) |
| 4 | 3-Hydroxybutyrate | $\gamma CH_3$, $\beta CH$, $\alpha CH_2$ | 1.20(d), 2.31(m), 2.41(m), 4.16(m) |
| 5 | Lactate | $CH_3$, CH | 1.33(d), 4.11(q) |
| 6 | Alanine | $\beta CH_3$, $\alpha CH$ | 1.48(d), 3.78(q) |
| 7 | Lysine | $\delta CH_2$ | 1.48(m), 1.73(m), 1.91(m), 3.03(t), 3.76(t) |
| 8 | Arginine | $\gamma CH_2$, $\beta CH_2$ | 1.78(m), 1.95(m) |
| 9 | GABA | $\alpha CH_2$, $\beta CH_2$, $\gamma CH_2$ | 1.91(m), 2.3(t), 3.02(t) |
| 10 | NAG | $CH_3$, $CH_{2,}$ $CH_2$, CH | 2.01(s), 2.32(m), 1.9-2.1(m), 4.2(m) |
| 11 | Glutamate | $\beta CH_2$, $\beta CH_2$, $\gamma CH_2$, $\alpha CH$ | 2.10(m), 2.14(m), 2.36(m), 2.50(m), 3.77(t) |
| 12 | Glutathione | S-$CH_2$, N-CH, N-$CH_2$, $CH_2$ | 2.14(m), 2.55(m), 2.95(m), 3.77(m), 4.56(t) |
| 13 | Acetoacetate | $CH_{3,}$ $CH_2$ | 2.28(s), 3.43(s) |
| 14 | Pyruvate | $CH_3$ | 2.37(s) |
| 15 | Succinate | $CH_2$ | 2.40(s) |
| 16 | Glutamine | $\beta CH_2$, $\gamma CH_2$, $\alpha CH$ | 2.47(m), 3.77(t) |
| 17 | Aspartate | $\beta CH_2$, α-CH | 2.68(m), 2.82(m), 3.91(m) |
| 18 | Citrate | 1/2$CH_2$, 1/2$CH_2$ | 2.67(d), 2.80(d) |
| 19 | Creatinine | N-$CH_3$, N-$CH_2$-CO | 3.01(s), 4.05(s) |
| 20 | Creatine | $CH_2$, $CH_3$ | 3.04(s), 3.93(s) |
| 21 | PCr | $CH_2$, $CH_3$ | 3.04(s), 3.93(s) |
| 22 | Choline | $N(CH_3)_3$, N-$CH_2$ | 3.25(s), 3.51(m) |
| 23 | OPC | $N(CH_3)_3$, N-$CH_2$, O-$CH_2$ | 3.21(s), 3.57(t), 4.16(t) |
| 24 | Acetylcholine | $N(CH_3)_3$, N-$CH_2$, O-$CH_{2,}$ $CH_3$ | 3.21(s), 3.67(t), 4.51(t), 2.1(s) |
| 25 | Taurine | $NH_2$-$CH_2$, $SO_3$-$CH_2$ | 3.26(t), 3.42(t) |
| 26 | Betaine | $N(CH_3)_3$, $CH_2$ | 3.27(s), 3.90(s) |
| 27 | Myo-inositol | CH | 3.27 (t), 3.53(dd), 3.62 (t), 4.05 (t) |
| 28 | Glycine | $CH_2$ | 3.57(s) |
| 29 | Glycerol | $CH_{2,}$ CH | 3.6(m), 3.8(m) |
| 30 | Ascorbate | $CH_2$, CH | 3.74(d), 3.76(d), 4.03(m), 4.52(d) |
| 31 | Serine | $CH_2$, CH | 3.85(m), 3.98(m) |
| 32 | Inosine | O-CH-N,N-CH=N, N-CH=N | 6.10 (d), 8.23 (s), 8.34 (s) |
| 33 | Uracil | CH=CH-N | 7.54(d), 5.79(d) |
| 34 | Adenosine | CH, CH | 8.25 (s), 8.34 (s) |
| 35 | AMP | N=CH-N, N=CH-N | 8.23 (s), 8.56 (s) |

| No. | Metabolite | Assignments | Chemical shift |
|---|---|---|---|
| 36 | Tyrosine | H3/H5, C5H/C6H | 3.06(m), 3.20(m), 3.94(m), 6.91(d), 7.20(d) |
| 37 | Tryptophan | CH=CH | 3.49(m), 4.06(m), 7.21(t), 7.29(t), 7.33(s), 7.55(d), 7.74(d) |
| 38 | Phenylalanine | CH=CH | 3.13(m), 3.28(m), 4.00(m), 7.33(m), 7.38(m), 7.43(m) |
| 39 | Xanthine | NH=CH-N | 7.95(s) |
| 40 | 3-Methylxanthine | NH=CH-N, N-$CH_3$ | 3.52(s), 8.02(s) |
| 41 | Hypoxanthine | NH=CH-N, N=CH-NH | 8.18(s), 8.20(s) |
| 42 | Fumarate | CH=CH | 6.53(s) |

Multiplicity: singlet (s), doublet (d), triplet (t), doublet of doublets (dd), quartets (q), multiplet (m).

**Supplementary Table S9** Calibration curve, correlation coefficient ($r^2$), test range and instrumental LOD, LOQ for berberine, baicalin and jasminoidin in methanol solution (n=6)

| Compounds | Calibration curve [a] | $r^2$ | Test range (mg/ml) | LOD [b] (ug/ml) | LOQ [c] (ug/ml) |
|---|---|---|---|---|---|
| berberine | y = 6862.7x + 33.746 | 0.9994 | 0.0202 - 1.01 | 0.524 | 0.975 |
| baicalin | y = 16998x - 9.2631 | 0.9992 | 0.01723- 0.8615 | 0.482 | 0.103 |
| jasminoidin | y = 6848.3x + 23.443 | 0.9991 | 0.01197- 0.5985 | 0.554 | 0.982 |

[a] y is the peak area in UV chromatograms monitored at 238 nm, 254 nm and 280 nm for berberine, baicalin, jasminoidin, respectively; x is the compound amount injected

[b] LOD refers to the limits of detection.

[c] LOQ refers to the limits of quantification.

**Supplementary Table S10** Precision, accuracy and recovery of berberine, baicalin and jasminoidin (n=6).

| Compounds | RSD (%) | | Accuracy (%) | | Recovery |
|---|---|---|---|---|---|
| | Intra-day | Intra-day | Inter-day | Inter-day | (Mean ± S.D., %) |
| berberine | 4.3 | 4.3 | -1.4 | -2.1 | 102.3 ± 8.4 |
| baicalin | 6.4 | 6.4 | 2.3 | -1.9 | 95.4 ± 5.7 |
| jasminoidin | 5.2 | 5.2 | -3.2 | 0.8 | 94.7 ± 6.4 |

**Supplementary Table S11** Calibration curve, correlation coefficient (r2), test range, weight cofficient and LLOQ for berberine, baicalin and jasminoidin in methanol solution (n=6)

| Samples | Compounds | Calibration curves [a] | $r^2$ | Liner range (ng/ml) | Weight cofficient | LLOQ [b] (ng/ml) |
|---|---|---|---|---|---|---|
| Plasma | berberine | y=0.002x-0.0013 | 0.9989 | 5-1000 | $1/x^2$ | 5 |
| | baicalin | y=0.0015x-0.0016 | 0.9967 | 5-1000 | $1/x^2$ | 5 |
| | jasminoidin | y=0.0014x-0.0012 | 0.9946 | 5-1000 | $1/x^2$ | 5 |
| Brain | berberine | y=0.0019x-0.00047 | 0.9938 | 2-1000 | $1/x^2$ | 2 |
| | baicalin | y=0.0018x+0.0039 | 0.9988 | 2-1000 | $1/x^2$ | 2 |
| | jasminoidin | y=0.002x-0.000282 | 0.9976 | 2-1000 | $1/x^2$ | 2 |

a y is the peak area in LC-QTOF-MSMS for berberine, baicalin, jasminoidin, respectively; x is the compound amount injected

b LLOQ refers to the lower limits of quantitation.

**Supplementary Table S12** Precision and accuracy of berberine, baicalin and jasminoidin for QC samples in rat plasma (n=6)

| Samples | Compounds | Concentration (ng/mL) | RSD (%) | | Accuracy (%) | |
|---|---|---|---|---|---|---|
| | | | Intra-day | Inter-day | Intra-day | Inter-day |
| Plasma | berberine | 4 | 5.4 | 4.7 | -2.4 | -1.9 |
| | | 800 | 2.3 | 3.8 | 1.3 | -2.9 |
| | | 1600 | 4.6 | 5.2 | -1.2 | 0.7 |
| | baicalin | 4 | 6.5 | 5.3 | -2.6 | -3.6 |
| | | 800 | 3.3 | 3.7 | 0.2 | -2.7 |
| | | 1600 | 6.2 | 4.6 | -0.9 | 2.5 |
| | jasminoidin | 4 | 4.3 | 3.6 | -3.2 | -1.7 |
| | | 800 | 2.4 | 3.7 | 0.3 | -1.8 |
| | | 1600 | 4.8 | 2.8 | -1.5 | 2.3 |
| Brain | berberine | 4 | 7.2 | 6.2 | -2.3 | -3.7 |
| | | 800 | 8.9 | 5.0 | 0.3 | -2.2 |
| | | 1600 | 4.7 | 4.7 | -0.8 | 1.6 |
| | baicalin | 4 | 3.4 | 6.3 | -3.2 | -0.7 |
| | | 800 | 4.2 | 7.3 | 0.6 | -1.6 |
| | | 1600 | 8.4 | 6.2 | -0.3 | 2.5 |
| | jasminoidin | 4 | 3.5 | 6.3 | -2.8 | -0.9 |
| | | 800 | 4.4 | 6.7 | 1.7 | -2.6 |
| | | 1600 | 5.6 | 4.8 | -1.4 | 3.1 |

**Supplementary Table S13** Recovery and matrix effect for berberine, baicalin and jasminoidin in rat plasma (n=6)

| Samples | Compounds | Nominal con. (ng/mL) | Recovery (Mean ± SD, %) | RSD (%) | Matrix effect (Mean ± SD, %) | RSD (%) |
|---|---|---|---|---|---|---|
| Plasma | berberine | 4 | 88.4 ± 4.4 | 5.4 | 99.0 ± 5.1 | 5.7 |
| | | 800 | 90.6 ± 6.2 | 4.7 | 100.1 ± 6.0 | 3.1 |
| | | 1600 | 91.4 ± 3.8 | 3.8 | 99.7 ± 25.2 | 2.6 |
| | baicalin | 4 | 87.6 ± 5.4 | 9.3 | 91.1 ± 7.1 | 5.3 |
| | | 800 | 89.3 ± 4.4 | 3.2 | 92.5 ± 4.0 | 3.7 |
| | | 1600 | 93.2 ± 9.6 | 6.2 | 91.1 ± 4.5 | 4.6 |
| | jasminoidin | 4 | 85.4 ± 5.2 | 8.9 | 89.6 ± 2.1 | 6.2 |
| | | 800 | 86.6 ± 4.2 | 7.3 | 88.7 ± 3.2 | 6.8 |
| | | 1600 | 85.4 ± 1.8 | 7.9 | 90.0 ± 2.4 | 3.3 |
| Brain | berberine | 4 | 87.3 ± 4.5 | 10.2 | 93.1 ± 5.3 | 6.9 |
| | | 800 | 91.7 ± 3.6 | 5.0 | 88.4 ± 2.7 | 6.0 |
| | | 1600 | 89.4 ± 4.1 | 4.7 | 91.6 ± 2.4 | 4.7 |
| | baicalin | 4 | 80.8 ±6.4 | 5.2 | 89.1 ± 3.3 | 7.5 |
| | | 800 | 80.1 ± 5.5 | 3.9 | 88.9 ± 3.2 | 4.3 |
| | | 1600 | 81.2±4.6 | 4.6 | 97.1 ± 3.2 | 6.3 |
| | jasminoidin | 4 | 88.1 ± 4.1 | 2.3 | 97.6 ± 4.5 | 4.8 |
| | | 800 | 90.1 ± 2.3 | 4.6 | 92.5 ± 3.1 | 5.1 |
| | | 1600 | 88.7 ± 2.7 | 5.2 | 88.9 ± 6.4 | 6.6 |

**Supplementary Table S14** Stability of berberine, baicalin and jasminoidin in plasma under various storage conditions (n=6)

| Compounds | Condition | Concentration (ng/mL) | | RSD | Accuracy |
|---|---|---|---|---|---|
| | | Added | Measured | (%) | (%) |
| | | 4 | 3.95 ± 0.06 | 2.3 | -1.2 |
| berberine | Short-term stability | 800 | 794 ± 13 | 2.4 | -0.9 |
| | | 1600 | 1589 ± 121 | 5.8 | -0.5 |
| | | 4 | 4.03 ± 0.02 | 0.9 | 0.7 |
| | Long-term stability | 800 | 797 ± 16 | 1.2 | -0.4 |
| | | 1600 | 1591 ± 113 | 5.7 | -0.6 |
| | | 4 | 3.89 ± 0.12 | 2.9 | -1.9 |
| | Freeze-thaw stability | 800 | 787 ± 17 | 2.0 | -1.2 |
| | | 1600 | 1582 ± 136 | 7.6 | -1.0 |
| | | 4 | 3.87 ± 0.18 | 4.7 | -3.3 |
| | Post-preparative stability | 800 | 774 ± 24 | 2.7 | -2.9 |
| | | 1600 | 1593 ± 126 | 7.0 | -0.4 |
| | | 4 | 4.00 ± 0.06 | 1.5 | -1.3 |
| baicalin | Short-term stability | 800 | 789 ± 8 | 1.5 | -0.7 |
| | | 1600 | 1581 ± 129 | 6.8 | -0.6 |
| | | 4 | 4.01 ± 0.03 | 0.5 | 0.8 |
| | Long-term stability | 800 | 792 ± 17 | 1.8 | -0.3 |
| | | 1600 | 1588 ± 110 | 6.3 | -0.5 |
| | | 4 | 3.82 ± 0.15 | 3.1 | -2.8 |
| | Freeze-thaw stability | 800 | 785 ± 14 | 1.9 | -1.4 |
| | | 1600 | 1592 ± 126 | 6.7 | -1.1 |
| | | 4 | 3.78 ± 0.22 | 7.4 | -2.3 |
| | Post-preparative stability | 800 | 784 ± 14 | 7.2 | -3.4 |
| | | 1600 | 1595 ± 123 | 5.0 | -0.6 |
| | | 4 | 3.92 ± 0.08 | 1.4 | -1.3 |
| jasminoidin | Short-term stability | 800 | 796 ± 4 | 1.6 | -0.5 |
| | | 1600 | 1582 ± 128 | 6.3 | -0.9 |
| | | 4 | 4.02 ± 0.01 | 0.9 | 0.7 |
| | Long-term stability | 800 | 791 ± 7 | 2.1 | -0.4 |
| | | 1600 | 1581 ± 129 | 5.6 | -0.6 |
| | | 4 | 3.90 ± 0.11 | 2.8 | -3.0 |
| | Freeze-thaw stability | 800 | 789 ± 11 | 2.2 | -1.6 |
| | | 1600 | 1582 ± 136 | 4.7 | -0.4 |
| | | 4 | 3.89 ± 0.11 | 3.9 | -1.3 |
| | Post-preparative stability | 800 | 784 ± 14 | 3.0 | -3.1 |
| | | 1600 | 1593 ± 126 | 5.7 | -0.7 |

**Supplementary Table S15** Stability of berberine, baicalin and jasminoidin in brain extracts under various storage conditions (n=6)

| Compounds | Condition | Concentration (ng/mL) | | RSD | Accuracy |
|---|---|---|---|---|---|
| | | Added | Measured | (%) | (%) |
| | | 4 | 3.85 ±0.14 | 2.5 | -2.3 |
| berberine | Short-term stability | 800 | 794 ±13 | 4.5 | -0.9 |
| | | 1600 | 1589 ±121 | 5.8 | -1.6 |
| | | 4 | 4.03 ±0.02 | 0.6 | 0.3 |
| | Long-term stability | 800 | 797 ±15 | 1.1 | -0.6 |
| | | 1600 | 1592 ±116 | 6.5 | -0.9 |
| | | 4 | 3.89 ±0.13 | 3.2 | -2.5 |
| | Freeze-thaw stability | 800 | 787 ±11 | 2.1 | -1.3 |
| | | 1600 | 1589 ±130 | 7.0 | -1.1 |
| | | 4 | 3.87 ±0.18 | 5.7 | -2.3 |
| | Post-preparative stability | 800 | 774 ±23 | 4.7 | -1.9 |
| | | 1600 | 1595 ±106 | 6.0 | -0.7 |
| | | 4 | 3.93 ±0.07 | 1.4 | -1.1 |
| baicalin | Short-term stability | 800 | 794 ±15 | 1.0 | -0.6 |
| | | 1600 | 1589 ±118 | 5.8 | -0.2 |
| | | 4 | 4.01 ±0.02 | 0.6 | 0.9 |
| | Long-term stability | 800 | 797 ±8 | 1.7 | -0.4 |
| | | 1600 | 1591 ±110 | 6.2 | -0.7 |
| | | 4 | 3.90 ±0.13 | 3.2 | -1.8 |
| | Freeze-thaw stability | 800 | 787 ±23 | 2.3 | -0.4 |
| | | 1600 | 1582 ±140 | 5.6 | -1.0 |
| | | 4 | 3.87 ±0.18 | 3.7 | -3.1 |
| | Post-preparative stability | 800 | 774 ±30 | 2.9 | -1.9 |
| | | 1600 | 1593 ±116 | 6.0 | -0.8 |
| | | 4 | 3.96 ±0.07 | 1.4 | -1.8 |
| jasminoidin | Short-term stability | 800 | 794 ±15 | 1.4 | -0.9 |
| | | 1600 | 1589 ±119 | 6.1 | -0.7 |
| | | 4 | 4.02 ±0.03 | 0.6 | 0.9 |
| | Long-term stability | 800 | 797 ±15 | 1.9 | -0.6 |
| | | 1600 | 1591 ±110 | 6.6 | -0.7 |
| | | 4 | 3.88 ±0.11 | 3.2 | -2.9 |
| | Freeze-thaw stability | 800 | 787 ±19 | 2.9 | -2.4 |
| | | 1600 | 1582 ±133 | 6.7 | -2.0 |
| | | 4 | 3.85 ±0.15 | 5.7 | -3.4 |
| | Post-preparative stability | 800 | 774 ±26 | 2.8 | -1.9 |
| | | 1600 | 1593 ±119 | 6.0 | -1.4 |

# Supplementary Figures

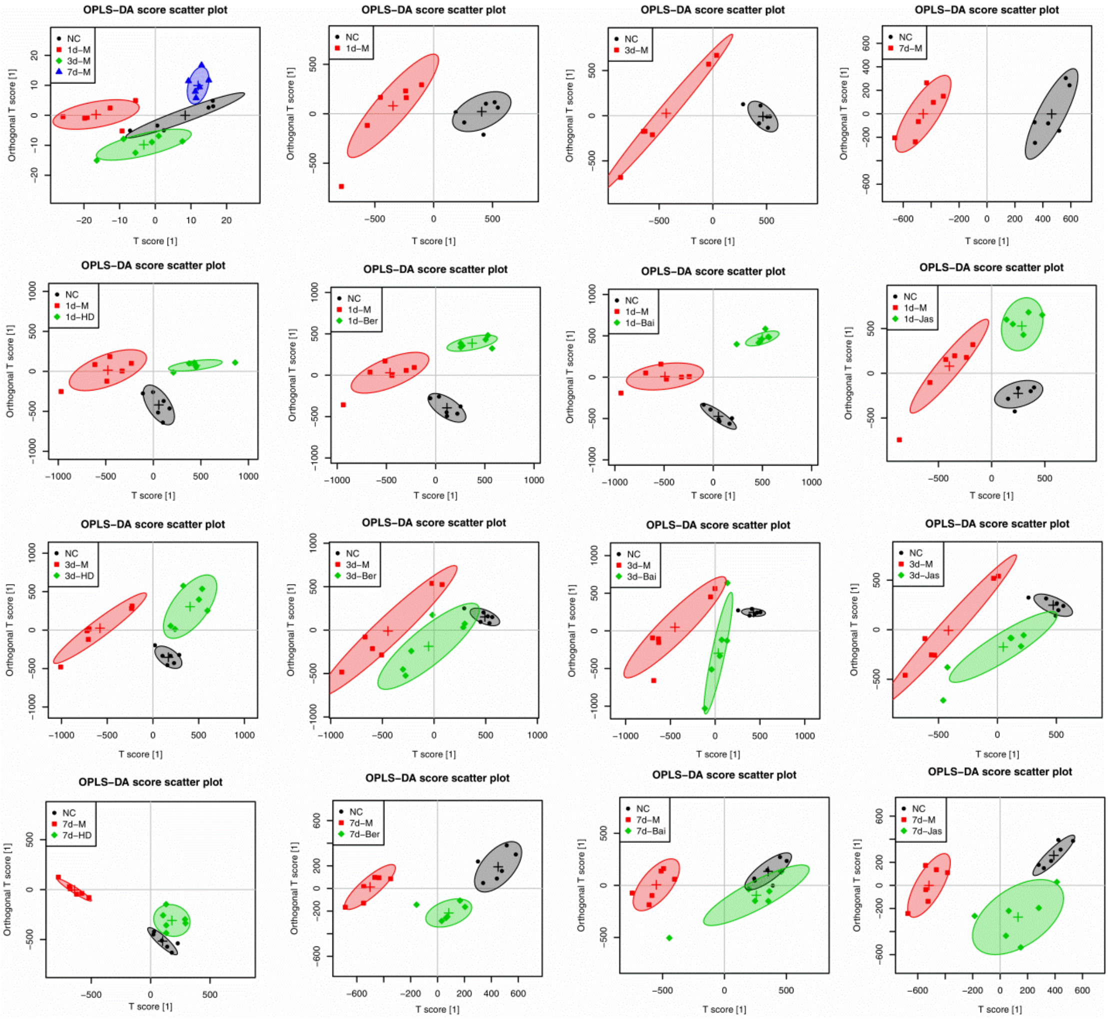


**Supplementary Figure S1** Score plots according to OPLS-DA analysis based on $^{1}$H NMR data from serum of all groups at each time point.

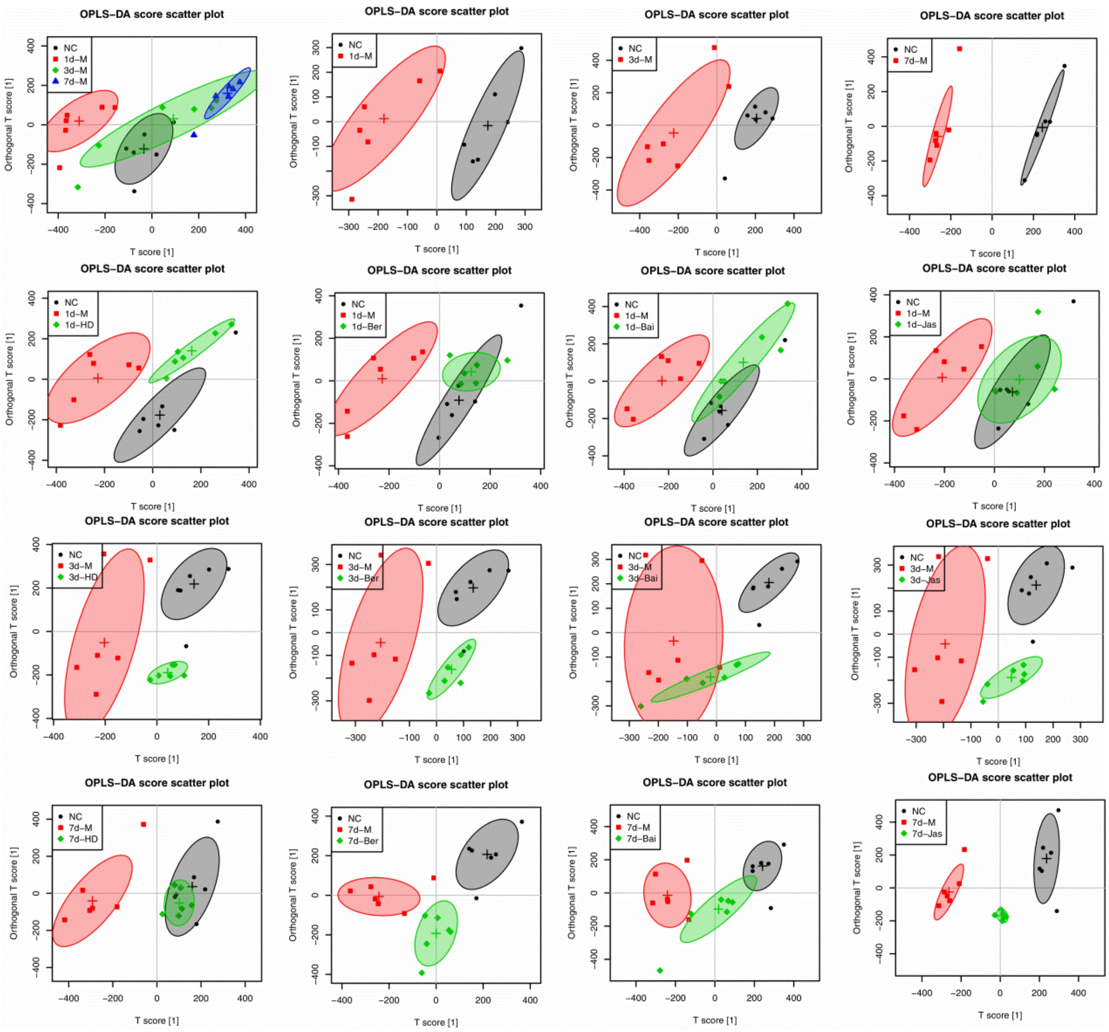

**Supplementary Figure S2** Score plots according to OPLS-DA analysis based on $^{1}$H NMR data from brain extracts of all groups at each time point.

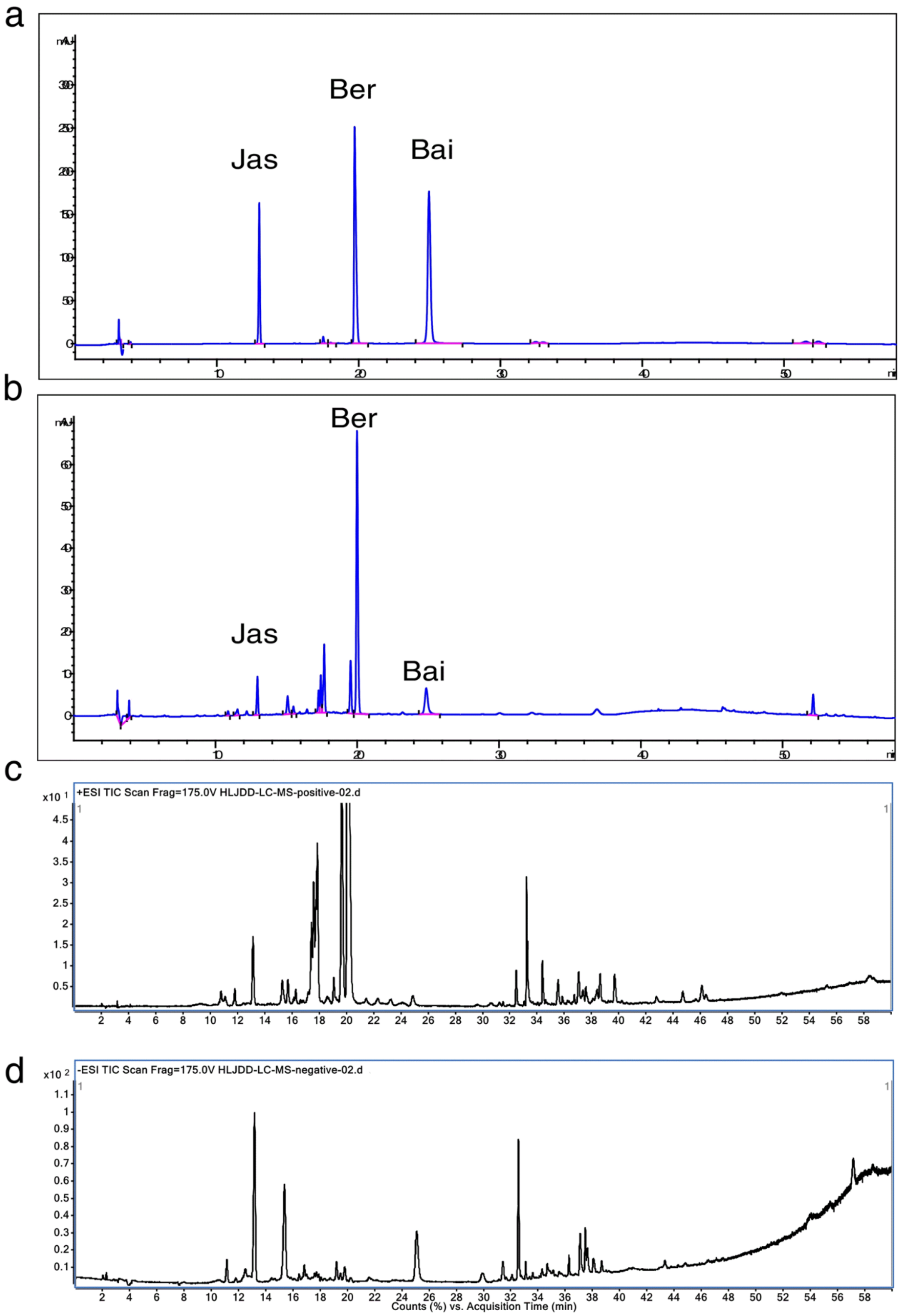


**Supplementary Figure S3** Profile of UV chromatograms and TIC chromatograms

(a) HPLC-UV chromatogram (0-60min) of berberine, geniposide and baicalin at 254 nm. (b) HPLC-UV chromatogram (0-60min) of total extract of Huang-Lian-Jie-Du Decoction (HLJDD) at

254 nm. (c) Total ion current (TIC) chromatogram (0-60 min) of HLJDD extract analyzed by HPLC-Q-TOF-MS in a positive ion mode. (d) TIC chromatogram (0-60 min) of HLJDD analyzed by HPLC-Q-TOF-MS in a negative ion mode.

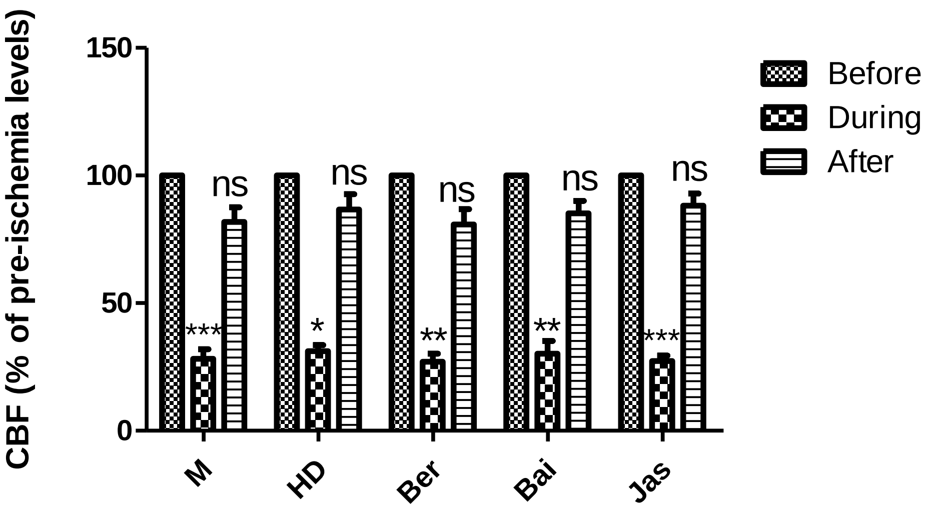


**Supplementary Figure S4** Quantitative analysis of regional cerebral blood flow (rCBF) in different groups. Data are expressed as mean ± S.D., n= 8. *: $p$ <0.05, **: $p$ <0.01, ***: $p$ <0.001 vs. pre-ischemia levels.

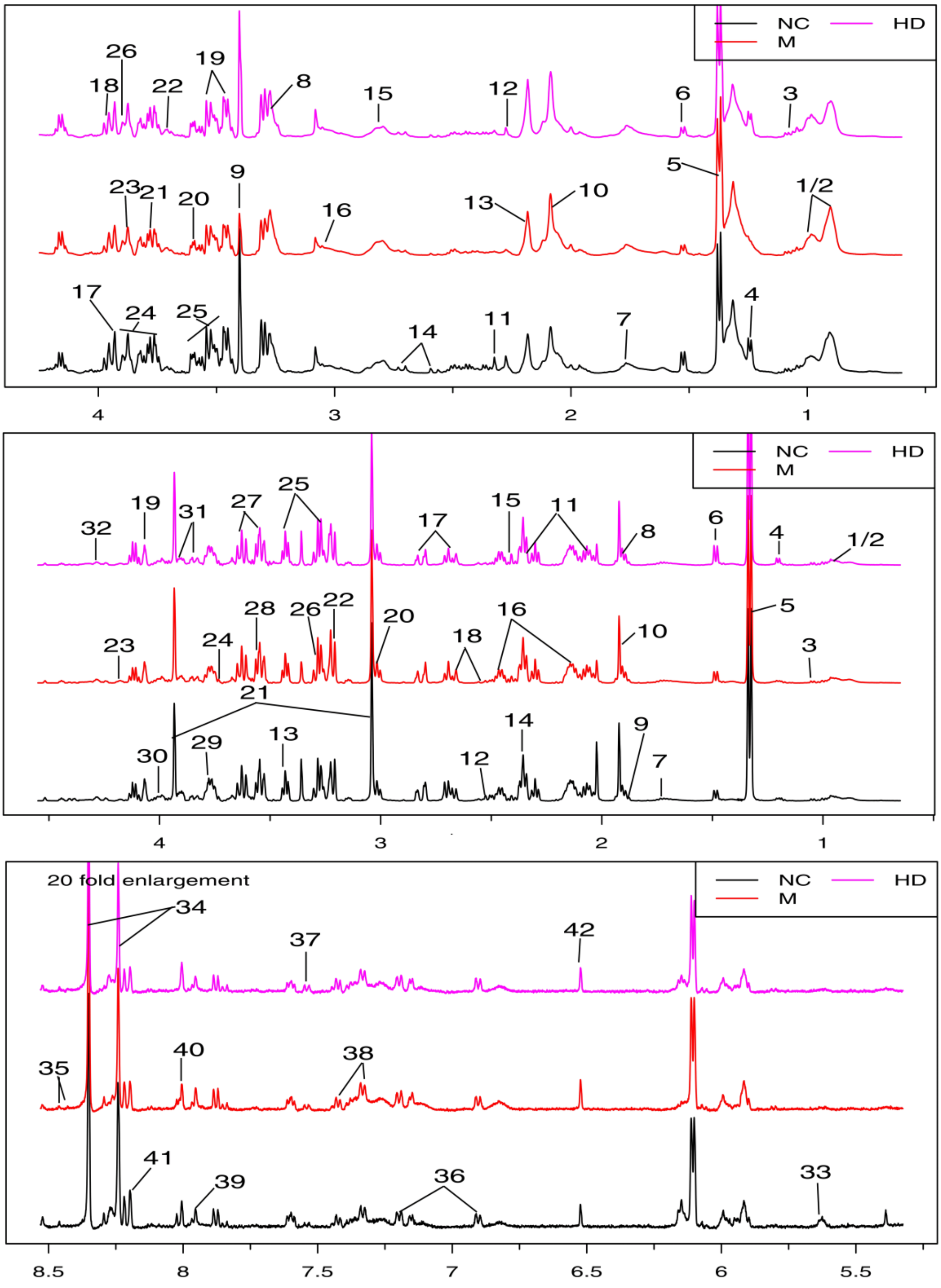


**Supplementary Figure S5** Typical 500 MHz $^{1}$H NMR spectra of serum and brain tissues

Typical 500 MHz $^{1}$H NMR spectra of serum (A) and cerebrum (B) obtained from the sham, the MCAO and the HD-treated groups. Metabolites in serum: 1, Isoleucine (Ile); 2, Leucine (Leu); 3, Valine (Val); 4, β-Hydroxybutyrate (3-HB); 5, Lactate (Lac); 6, Alanine (Ala); 7, Lysine (Lys); 8, Arginine (Arg); 9, Acetoacetate (Acet); 10, Glutamate (Glu); 11, Pyruvate (Pyr); 12, Succinate

(Suc); 13, Glutamine (Gln); 14, Citrate (Cit); 15, Nicotinamide adenine dinucleotide phosphate (NADPH); 16, Cystine (Cys); 17, Creatine (Cr); 18, Trimetlylamine oxide (TMAO); 19, Taurine (Tau); 20, Glycine (Gly); 21, Glycerol (Gyo); 22, Acetylcholine (Ach); 23, Betaine (Bet); 24, α-Glucose (α-Glc); 25, β-Glucose (β-Glc); 26, O-phosphocholine (OPC).

Metabolites in brain tissues: 1, Isoleucine (Ile); 2, Leucine (Leu); 3, Valine (Val); 4, β-Hydroxybutyrate (3-HB); 5, Lactate (Lac); 6, Alanine (Ala); 7, Lysine (Lys); 8, Arginine (Arg); 9, γ-amino-butyrate (GABA); 10, N-acetyl-glutamate (NAG); 11, Glutamate (Glu); 12, Glutathione (GSH); 13, Acetoacetate (Ace); 14, Pyruvate (Pyr); 15, Succinate (Suc); 16, Glutamine (Gln); 17, Asparate (Asp); 18, Citrate (Cit); 19, Creatinine (Cre); 20, Creatine (Cr); 21, Phosphocreatine (PCr); 22, choline (Cho); 23, O-phosphocholine (OPC); 24, Acetylcholine (Ach); 25, Taurine (Tau); 26, Betaine (Bet); 27, Myo-inositol (Myo); 28, Glycine (Gly); 29, Glycerol (Gyo); 30, Ascorbate (Asc); 31, Serine (Ser); 32, Inosine (Ino); 33, Uracil (Ura); 34, Adenosine (Ade); 35, Adenosine monophosphate monophosphate (AMP); 36, Tyrosine (Tyr); 37, Tryptophan (Trp); 38, Phenylalanine (Phe); 39, Xanthine (Xan); 40, 3-Methylxanthine (3-MX); 41, Hypoxanthine (Hyp); 42, Fumarate (Fum).

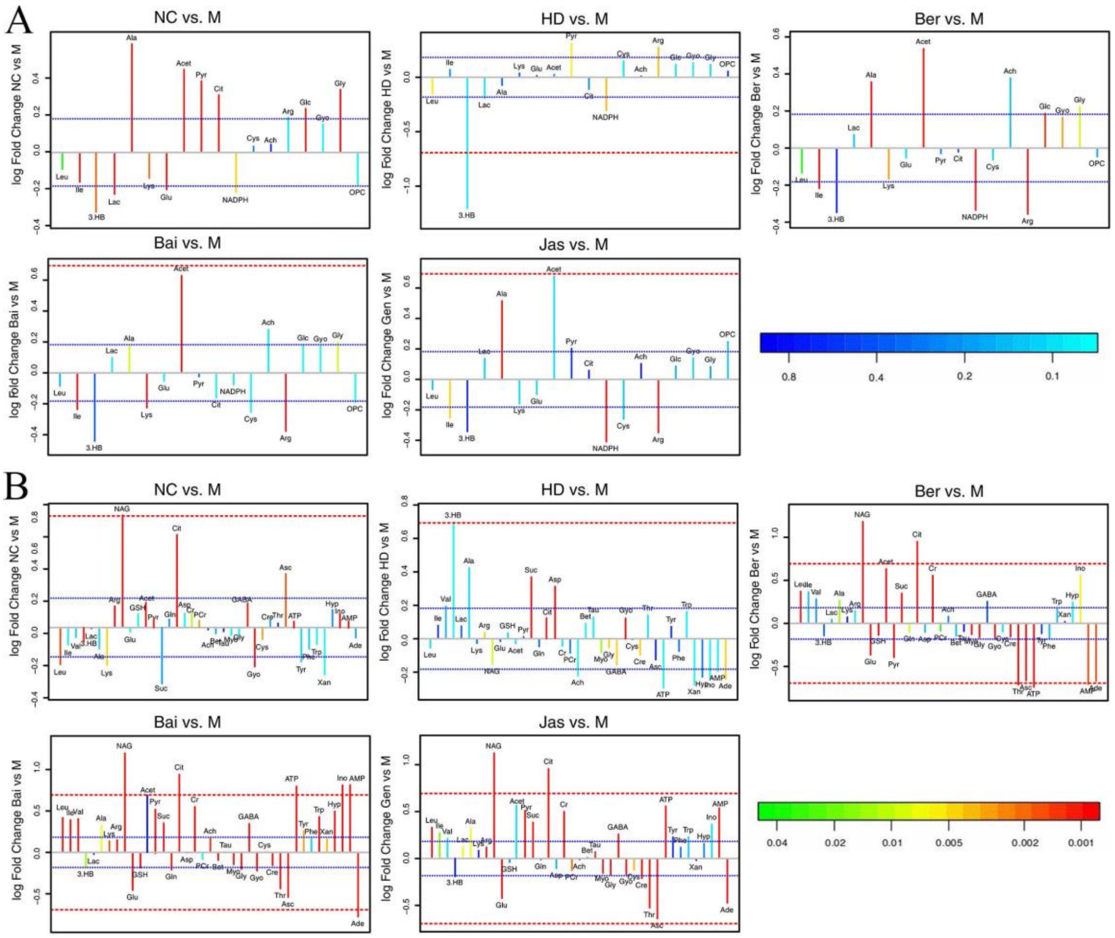


**Supplementary Figure S6** Fold change plots color-coded with p-values adjusted by Benjamini-Hochberg method indicating significance of altered metabolites in serum (A) and brain extracts (B). The blue and red dashed lines represented variations of 20% and 100%, respectively.

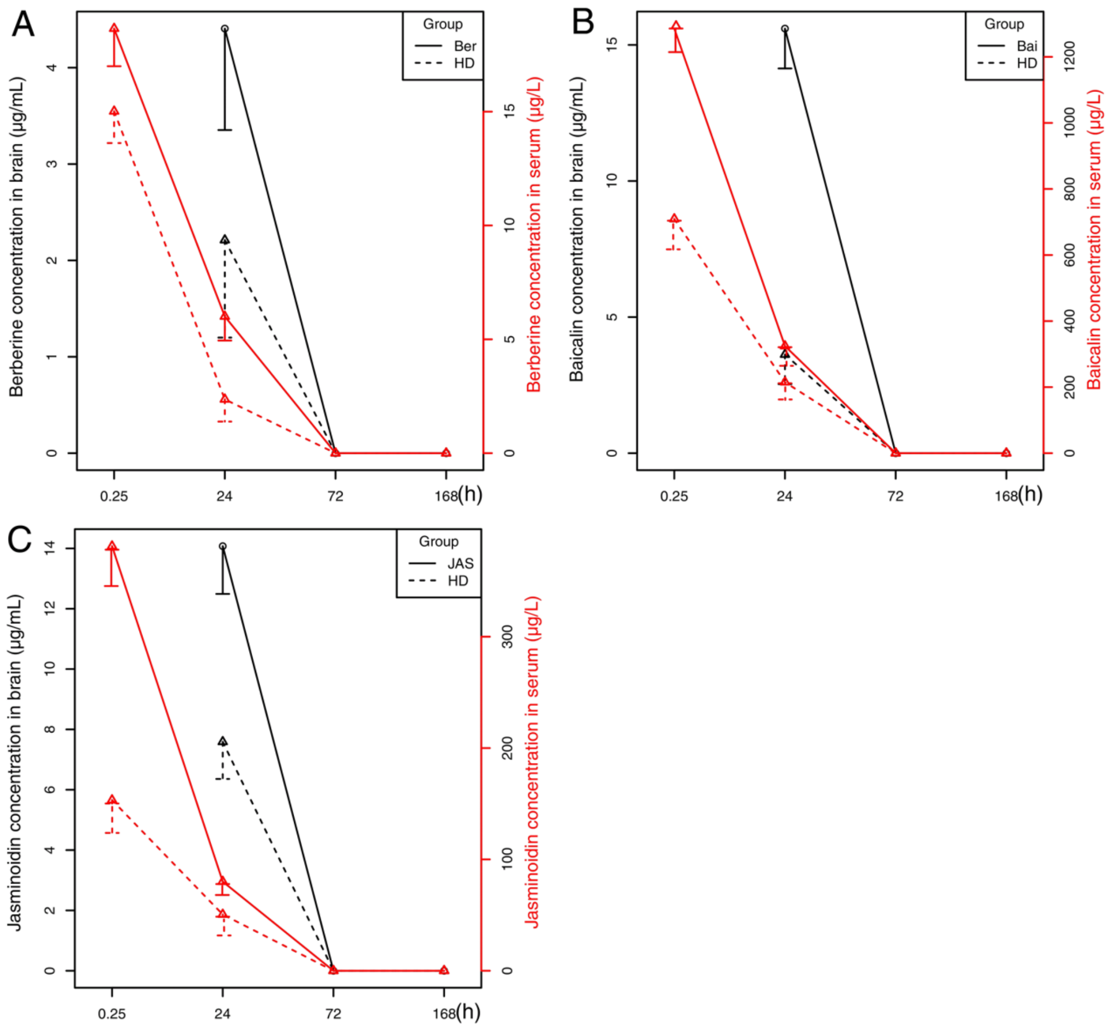


**Supplementary Figure S7** Brain and plasma concentration versus time profiles of berberine, baicalin and jasminoidin after administration in rats. Data represents mean ±S.D. (n = 6).

# Supplementary References


[1] J. Xia, N. Psychogios, N. Young, and D.S. Wishart, "MetaboAnalyst: a web server for metabolomic data analysis and interpretation," *Nucleic acids research*, vol. 37, no. suppl 2, pp. W652-W660, 2009.

[2] Y. Benjamini, and Y. Hochberg, "Controlling the false discovery rate: a practical and powerful approach to multiple testing," *Journal of the Royal Statistical Society. Series B (Methodological)*, vol., pp. 289-300, 1995.

[3] S.R. Kim, D.I. Kim, M.R. Kang, K.S. Lee, S.Y. Park, J.S. Jeong, and Y.C. Lee, "Endoplasmic reticulum stress influences bronchial asthma pathogenesis by modulating nuclear factor kappaB activation," *Journal Of Allergy And Clinical Immunology*, vol. 132, no. 6, pp. 1397-1408, 2013.

[4] A. Arocho, B. Chen, M. Ladanyi, and Q. Pan, "Validation of the 2-ΔΔCt calculation as an alternate method of data analysis for quantitative PCR of BCR-ABL P210 transcripts," *Diagnostic Molecular Pathology*, vol. 15, no. 1, pp. 56-61, 2006.
